# Dynamic Protein Phosphorylation in *Streptococcus pyogenes* during Growth, Stationary Phase, and Starvation

**DOI:** 10.3390/microorganisms12030621

**Published:** 2024-03-20

**Authors:** Stefan Mikkat, Michael Kreutzer, Nadja Patenge

**Affiliations:** 1 Core Facility Proteome Analysis, Rostock University Medical Center, 18057 Rostock, Germany; 2 Medical Research Center, Rostock University Medical Center, 18057 Rostock, Germany; michael.kreutzer@med.uni-rostock.de; 3 Institute of Medical Microbiology, Virology and Hygiene, Rostock University Medical Center, 18057 Rostock, Germany

**Keywords:** *Streptococcus pyogenes*, phosphoproteome, phosphorylation site, phosphopeptide enrichment, PASTA kinase, growth phase, starvation, proteomics, label-free quantification

## Abstract

Phosphorylation of proteins at serine, threonine, and tyrosine residues plays an important role in physiological processes of bacteria, such as cell cycle, metabolism, virulence, dormancy, and stationary phase functions. Little is known about the targets and dynamics of protein phosphorylation in *Streptococcus pyogenes*, which possesses a single known transmembrane serine/threonine kinase belonging to the class of PASTA kinases. A proteomics and phosphoproteomics workflow was performed with *S. pyogenes* serotype M49 under different growth conditions, stationary phase, and starvation. The quantitative analysis of dynamic phosphorylation, which included a subset of 463 out of 815 identified phosphorylation sites, revealed two main types of phosphorylation events. A small group of phosphorylation events occurred almost exclusively at threonine residues of proteins related to the cell cycle and was enhanced in growing cells. The majority of phosphorylation events occurred during stationary phase or starvation, preferentially at serine residues. PASTA kinase-dependent cell cycle regulation processes found in related bacteria are conserved in *S. pyogenes*. Increased protein phosphorylation during the stationary phase has also been described for some other bacteria, and could therefore be a general feature in the physiology of bacteria, whose functions and the kinases involved need to be elucidated in further analyses.

## 1. Introduction

Protein phosphorylation is one of the most important and best studied post-translational modifications involved in the regulation of various biological processes [1]. Phosphorylation alters the physicochemical properties of a protein and can thereby control cellular processes, for example, by inducing conformational changes in the active site of the protein or regulating protein–protein interactions [2]. The opposing action of phosphorylating kinases and dephosphorylating phosphatases enables rapid and precise regulation.

In bacteria, signal transduction by two-component systems involving phosphorylation of histidine and arginine residues has traditionally been considered to be almost the only form of protein phosphorylation. However, during the last two decades it has become increasingly evident that phosphorylation at serine, threonine, and tyrosine residues plays vital roles in several physiological processes, such as cell cycle and cell wall synthesis, bacterial metabolism, virulence, dormancy, sporulation, and stationary phase functions [1].

Enrichment of phosphopeptides using immobilized metal affinity chromatography (IMAC) or TiO_2_ affinity chromatography [3] with subsequent LC-MS/MS analysis has been established as the method of choice for site-specific serine/threonine/tyrosine bacterial phosphoproteomics. Early pioneering projects using this approach typically identified nearly one hundred phosphorylation sites in the phosphoproteomes of *Bacillus subtilis* [4], *Escherichia coli* [5], *Lactococcus lactis* [6], and other bacteria [7]. This number increased dramatically to several thousand with technological advances in qualitative and quantitative mass spectrometry, data management, and phosphopeptide sample preparation [8,9,10]. Despite these advances, there are major gaps in our knowledge of the functions of protein phosphorylation in bacteria. The kinases/phosphatases that regulate the hundreds and thousands of identified phosphorylation events as well as the full range of native substrates of known kinases/phosphatases are largely unknown. Functional redundancy of different kinases and/or substrate promiscuity complicate their elucidation [2]. In addition, there are no unique sequence motifs for bacterial kinase targets.

Bacterial phosphorylation at serine and threonine residues is mainly catalyzed by eukaryotic-like Ser/Thr kinases (eSTKs) that possess a catalytic domain sharing a common fold with eukaryotic protein kinases. Ubiquitous in *Actinobacteria* and *Firmicutes* are transmembrane eSTKs composed of a cytoplasmic catalytic kinase domain linked by a transmembrane segment to an extracellular domain consisting of a variable number of PASTA (penicillin-binding protein and serine/threonine-associated) repeats [2,11]. Binding of ligands, such as beta-lactam compounds and cell wall fragments, to the PASTA domains triggers the kinase activity [12]. PASTA kinase activity affects the virulence of streptococci and other *Firmicutes* in animal models (reviewed by [13]). Tyrosine phosphorylation is performed by bacterial idiosyncratic tyrosine kinases (BY-kinases) [14,15] and by the family of ubiquitous bacterial kinases (UbK) [16]. In addition, atypical and novel kinases contribute to the diversity of bacterial systems for phosphorylation of serine, threonine, and tyrosine [15,17,18].

*Streptococcus pyogenes* is a host-adapted human pathogen responsible for a wide range of clinical manifestations, including asymptomatic infection, superficial self-limiting infections, invasive life-threatening diseases, and autoimmune sequelae. Together, the global burden of disease caused by *S. pyogenes* is high [19]. The course of an infection depends on the appropriate expression of specific virulence factors and on successful adaption to the host environment [20]. Control of virulence gene expression by stand-alone transcription factors and two-component systems are known to play a role in virulence determination in *S. pyogenes* [21,22]. An additional level of bacterial gene expression control is provided by small regulatory RNAs [23,24]. Furthermore, protein phosphorylation could play a crucial role in the pathogenicity of *S. pyogenes*, as already known for other bacteria [13].

*S. pyogenes* possesses one PASTA kinase and its cognate protein phosphatase, which are products of cotranscribed genes. In *S. pyogenes* M1, the kinase SP-STK was autophosphorylated in vitro on threonine residues, and was dephosphorylated by the phosphatase SP-STP. Reversible phosphorylation by SP-STK/SP-STP affected cell division and septation, shape, size, expression and surface display of certain proteins, and interaction of *S. pyogenes* with eukaryotic cells [25]. Hereafter, the PASTA kinase (Spy49_1257c) and the cognate phosphatase (Spy49_1259c) of *S. pyogenes* M49 will be referred to as SP-STK and SP-STP, respectively, consistent with the proteins from *S. pyogenes* M1 [25].

Compared to *Streptococcus pneumoniae*, little is known about the role of protein phosphorylation in *S. pyogenes*. A widely used approach in bacterial phosphoproteomics is the identification of kinase targets by comparing the phosphorylation profiles of wild-type and kinase mutant strains, usually investigating a single growth phase. In this study, we took a different approach by comparing phosphorylation dynamics during specific growth phases and nutritional conditions to identify collectively regulated phosphorylation sites that could indicate their involvement in specific physiological processes. During our research, the first phosphoproteome of *S. pyogenes* was published [26] listing 449 phosphorylation sites from a single growth condition of the M1 wild type. In this study, we report a comprehensive phosphoproteomic analysis of *S. pyogenes* M49 during growth, stationary phase, and starvation, in which 815 phosphorylation sites were identified and the phosphorylation dynamics of 463 sites were quantitatively analyzed.

## 2. Materials and Methods

### 2.1. Bacterial Culture Conditions

**Figure 1:**
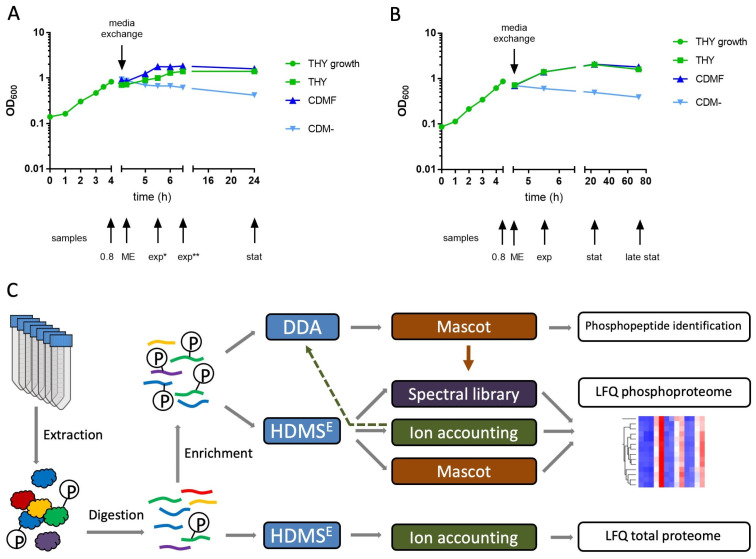
Bacterial growth and proteomics workflow. (**A**,**B**) *S. pyogenes* was grown in THY to an OD_600_ = 0.8 (green circles). For media exchange (ME), bacterial cultures were centrifuged and pellets were suspended in either THY (THY, green squares), CDM without carbon source (CDM-, light blue triangles), or CDM with 1% fructose (CDMF, dark blue triangles). Sample collection is indicated by arrows. exp*: exponential growth phase (CDMF, one doubling), exp**: exponential growth phase (THY, one doubling), stat: stationary phase, late stat: late stationary phase. (**A**) In the first experiment, *S. pyogenes* was cultured for 24 h. (**B**) In the second experiment, *S. pyogenes* was cultured for 72 h. (**C**) Experimental workflow for proteomic and phosphoproteomic nano LC-MS/MS analysis using a Synapt G2-S mass spectrometer and the analysis software Progenesis QI for proteomics, version 4.1 (Nonlinear Dynamics, Newcastle upon Tyne, UK). For label-free quantification (LFQ) of the total proteome, tryptic digests of the extracted proteins were subjected to data-independent HDMS^E^ acquisition. The ion accounting algorithm implemented in Progenesis was used for peptide and protein identification. Enriched phosphopeptides were subjected to both data-dependent (DDA) and HDMS^E^ acquisition. Peak lists from the DDA measurements were exported to Mascot for identification. The HDMS^E^ data were subjected to peptide identification by Mascot, ion accounting and comparison with a spectral library assembled from phosphopeptides identified in the DDA/Mascot approach (indicated by the brown arrow). For some DDA acquisitions, precursor selection was based on an inclusion list generated from the results of the HDMS^E^/ion accounting approach (indicated by the dashed arrow).

*S. pyogenes* serotype M49 strain 591 [27,28] was cultured in Todd-Hewitt broth supplemented with 0.5% yeast extract (THY; Oxoid, Thermo Fisher Scientific, Darmstadt, Germany) at 37 °C under a 5% CO_2_/20% O_2_ atmosphere. Overnight cultures were diluted 1:20 in 40 mL of THY and grown to an OD_600_ = 0.8. For media exchange, the bacteria were centrifuged at 4000× *g* for 20 min. The pellet was suspended in either THY or chemically defined medium [29] without carbon source (CDM-) or with 1% fructose (CDMF). The cells were harvested at different time points as indicated (Figure 1 and Figure S1). The culture was centrifuged at 4000× *g* for 10 min at 4 °C. The pellet was washed in cold PBS, subsequently shock frozen in liquid nitrogen, and stored at −80 °C.

### 2.2. Sample Preparation for Proteomics

Ice-cooled bacterial cells were disrupted with glass beads using Precellys 24 homogenizer (peqLab Biotechnologie GmbH, Erlangen, Germany) in non-denaturing buffer containing 10 mM Tris/HCl, pH 7.4, 138 mM NaCl, 2.7 mM KCl, and 1 mM MgCl_2_. Immediately thereafter, Tris-HCl, pH 8.0 and sodium deoxycholate (SDC) were added from stock solutions to obtain final concentrations of 50 mM Tris-HCl and 2% SDC. The samples were incubated at 95 °C for 5 min before the protein extracts were aspirated from the glass beads, and further sonicated for 10 min using a bath sonicator. The raw cell extract containing cell debris was used for the subsequent sample processing steps including proteolytic digestion. The protein concentration was measured using the Bio-Rad protein assay (Bio-Rad, Munich, Germany). Reduction and alkylation was performed for 15 min at 37 °C after addition of 1/10 volume reduction/alkylation reagent containing 100 mM tris(2-carboxyethyl)phosphine hydrochloride (TCEP) and 400 mM 2-chloroacetamide (CAM) [30]. Next, methanol/chloroform precipitation was performed as previously described [31]. The precipitate was dissolved in digestion buffer composed of 100 mM Tris-HCl, pH 8.0, 1% SDC, 5 mM TCEP, and 20 mM CAM. Sequencing grade trypsin (Promega GmbH, Walldorf, Germany) was added to obtain an enzyme/protein ratio of approximately 1:100, and digestion was performed at 37 °C for about 16 h. Afterwards, the cell debris was removed by centrifugation. The supernatant was acidified to a final concentration of 0.7% trifluoroacetic acid (TFA), mixed vigorously, and the precipitated SDC was pelleted by centrifugation at 13,000 rpm for 10 min. Finally, the peptide solutions were desalted with OASIS HLB 1cc 30 mg Vac Cartridges (Waters, Manchester, UK), and the eluate was fivefold concentrated using a centrifugal evaporator. Peptide concentrations were measured using the Invitrogen Qubit protein assay kit (Thermo Fisher Scientific, Darmstadt, Germany).

### 2.3. Phosphopeptide Enrichment

Peptide amounts of 100 µg or 250 µg were evaporated to dryness using a centrifugal evaporator. MagReSyn TiO_2_ and MagReSyn Ti-IMAC hyperporous magnetic microparticles (ReSyn Biosciences, Edenvale, Gauteng, South Africa) were used as a mixture to take advantage of combined enrichment chemistries [32]. The compositions of the loading buffer (1 M glycolic acid in 80% acetonitrile (ACN) and 5% TFA), wash buffer 1 (80% ACN, 1% TFA), wash buffer 2 (10% ACN, 0.2% TFA), and elution buffer (1% NH_4_OH) corresponded to the manufacturer’s recommendations for use with MagReSyn TiO_2_ microparticles. For the enrichment of phosphopeptides from one sample, 15 µL of Ti-IMAC and 7.5 µL of TiO_2_ were mixed and equilibrated with loading buffer. The dried peptides were dissolved in 200 µL loading buffer, freed from insoluble material by centrifugation, and transferred to the pellet of equilibrated microparticles. The phosphopeptides were bound to the microparticles during an incubation of 20 min at room temperature with constant mixing. Then, three consecutive washes of two min each were performed with 100 µL of loading buffer, wash buffer 1, and wash buffer 2. Bound phosphopeptides were eluted from the microparticles with 80 µL of elution buffer with gentle mixing for 10 min. The eluate was transferred to a protein LoBind tube (Eppendorf, Hamburg, Germany) containing 20 µL of 10% formic acid (FA). The elution was repeated with 80 µL of elution buffer for 5 min, and both eluates were pooled and frozen. Subsequently, the samples were evaporated to near dryness using a centrifugal evaporator. Desalting of the phosphopeptides was performed on StageTips containing one C18 disk, as previously described [33]. Finally, phosphopeptides were dissolved in 20 µL of 2% ACN, 0.1% FA.

### 2.4. Mass Spectrometry

LC-MS analyses were carried out using a nanoAcquity UPLC system (Waters, Manchester, UK) coupled to a Waters Synapt G2-S mass spectrometer via a NanoLockSpray ion source as previously described [34], with a gradient time for chromatographic separation of 90 min. For the analysis of the total proteome, 70 ng of peptides were injected according to the results of the Invitrogen Qubit protein assay supplemented with 40 fmol of Hi3 Phos B standard for protein absolute quantification (Waters, Manchester, UK). For analyses of the phosphopeptides, typically 10% of the enriched sample was used.

For both the total proteome and phosphoproteome measurements, the Synapt G2-S instrument was operated in data-independent mode with ion mobility separation as an additional dimension of separation (referred to as HDMS^E^). By executing alternate scans at low and elevated collision energies of each 0.6 s, information on precursor and fragment ions was acquired, respectively [34,35]. Either duplicate or triplicate measurements were performed, depending on the experiment. Measurements of phosphoproteomes were additionally conducted in data-dependent mode (DDA). The typical parameters used were as follows. Following MS survey scans of 0.2 s, the instrument was switched to MS/MS acquisition if the intensity of individual ions exceeded the threshold of 50,000 counts per second. Up to three ions with charge states between 2+ and 4+ were selected from a single MS survey scan. The MS/MS scan rate was set to 0.2 s. The instrument returned to MS survey scan if the TIC threshold of 600,000 counts was reached, or after 2.1 s of MS/MS acquisition. Dynamic exclusion of already selected precursor ions was set to 12 s. In addition, inclusion lists generated from HDMS^E^ analyses of the respective experiments were used for some DDA acquisitions, whereby only precursor ions from the inclusion list were selected for MS/MS.

### 2.5. Data Processing, Protein Identification and Quantification

#### 2.5.1. Total Proteome

Progenesis QI for proteomics version 4.1 (Nonlinear Dynamics, Newcastle upon Tyne, UK) was used for raw data processing, protein identification, and label-free quantification, as described previously [34]. Proteins were identified using a database of 1701 protein sequences from *S. pyogenes* serotype M49 strain NZ131 (UniProt release 2021_02), appended with the sequences of rabbit phosphorylase B (P00489) and porcine trypsin. Proteins were quantified by the absolute quantification Hi3 method using Hi3 Phos B Standard (Waters, Manchester, UK) as reference [36]. Results were given as fmol on column.

#### 2.5.2. Phosphoproteome

Raw data from data-independent (HDMS^E^) acquisitions were processed with Progenesis QI for proteomics. Then, three different strategies for peptide and protein identification were applied to the same data: (i) ion accounting search, (ii) Mascot search, and (iii) spectral library search.

For identification using the ion accounting algorithm implemented in Progenesis, a database containing 1701 protein sequences from *S. pyogenes* serotype M49 strain NZ131 (UniProt release 2021_02) was used. Two missing cleavage sites were allowed, carbamidomethylation of cysteine residues was set as fixed modification, and methionine oxidation, asparagine deamidation, phosphorylation of serine, threonine, and tyrosine residues, as well as phosphoglyceryl modification of lysine residues were considered as variable modifications. The false discovery rate was set to 1%. Peptides were required to be identified by at least five fragment ions. Subsequently, the peptide ion data were filtered to retain only peptide ions that met the following criteria: (i) identified in at least two samples within the dataset; (ii) minimum ion score of 5.5; (iii) mass error below 13.0 ppm, and (iv) at least 8 amino acid residues in length. Identifications based on charge state deconvolution were removed. Deamidation of asparagine was only accepted if the asparagine residue was followed by glycine [37]. Subsequently, the fragment spectra of all remaining phosphopeptides were checked manually for plausibility of identification.

To identify HDMS^E^ data with the Mascot search engine, peak lists in mascot generic format (MGF) were generated in Progenesis. The number of exported fragment ions per spectrum was limited to 80, and deisotoping and charge deconvolution was enabled. The MGF files were searched against the *S. pyogenes* database using Mascot (version 2.6.2) applying the same enzyme specificity, with fixed and variable modifications used for identification with the ion accounting algorithm. The peptide mass tolerance was set to 13 ppm and the fragment mass tolerance to 0.02 Da. The significance threshold was adjusted to *p* < 0.01. Resulting protein false positive rates were between zero and 0.44% for the different experiments. The results data were imported into Progenesis and mapped to the peptide features. Then, phosphopeptides identified in only one sample of the data set were deleted. Deamidation of asparagine was only accepted if the asparagine residue was followed by glycine. The confidence of phosphorylation site localization was assessed using the Mascot Delta Score [38]. The maximum and mean Delta Score was calculated for multiple mass spectra belonging to the same feature. The maximum delta score was used to generate a list of all phosphopeptides, while the mean delta score was used to select phosphopeptides for quantitative analysis. We applied a threshold of 75% to avoid the exclusion of informative phosphorylation sites, e.g., from the PASTA kinase SP-STK, but manually validated the localization of phosphorylation sites with a localization probability of less than 95%.

To search HDMS^E^ data against a spectral library, spectral libraries were created in Progenesis with phosphopeptides that were unambiguously identified by DDA of the respective samples. Spectral library searches were performed using a peptide mass tolerance of 13 ppm, a fragment mass tolerance of 20 ppm, and a retention time window of 0.5 min. At least five matching fragments per peptide were required. Since it turned out that the positional variants of phosphopeptides could not be reliably distinguished in the spectral library search, the results of the spectral library search were only used if they were confirmed by the Mascot search. The ion accounting, Mascot, and spectral library search results of the HDMS^E^ data were further processed using Microsoft Office 2016.

Raw data from data-dependent (DDA) acquisitions were also processed with Progenesis QI for proteomics. Export of peak lists, peptide identification using the Mascot search engine, and further processing of search results were performed as described for the HDMS^E^ data. The mass spectrometry proteomics data have been deposited to the ProteomeXchange Consortium via the PRIDE [39] partner repository with the dataset identifiers PXD044423 and 10.6019/PXD044423. Unsupervised hierarchical clustering of protein level-normalized phosphorylation site abundances was performed using the Interactive CHM Builder [40] at https://build.ngchm.net/NGCHM-web-builder/, accessed on 9 August 2022. Cluster analysis of z-transformed rows was carried out using the Euclidean distance metric and average agglomeration method. Venn diagrams were calculated using Venny 2.1.0 at https://bioinfogp.cnb.csic.es/tools/venny/index.html, accessed on 1 July 2022.

## 3. Results

### 3.1. Experimental Rationale

This study was designed to provide initial insights into targets and dynamics of protein phosphorylation in *S. pyogenes*. For this purpose, bacteria were grown to late exponential phase in THY broth, then transferred to three different culture media and harvested at different time points. We used rich THY broth containing 0.2% glucose providing optimal growth conditions; chemically defined medium containing 1% fructose (CDMF) to induce growth on a single carbon source; and chemically defined medium without a carbon source (CDM-) to provoke starvation. In the first experiment, we found that the number of phosphorylation sites and the overall degree of protein phosphorylation increased sharply in the stationary growth phase and under starvation conditions. Therefore, the cultivation time was extended from 24 h to 72 h in the second experiment (Figure 1). A third experiment utilizing only THY broth was designed to find out whether the phosphorylation pattern changed within 40 min after the bacteria were transferred from the extended stationary phase to fresh medium (Figure S1). In total, proteomes and phosphoproteomes of 29 bacterial cultures were included in this study.

### 3.2. The Proteome during Growth in Different Culture Media

Quantitative data of the proteome were collected primarily to allow normalization of phosphopeptide amounts with the corresponding protein amounts, and are therefore only briefly presented here. Between 930 and 972 proteins identified by at least two unique peptides were quantified in the three experiments. There were 872 proteins that were common to all experiments, while in total, 1038 proteins were found, corresponding to 61% of the predicted proteins of *S. pyogenes*. The numbers of common and total proteins increased to 936 and 1121, respectively, when proteins whose identification was based on a single peptide were included (Table S1, Figure S2).

The levels of many proteins increased during the stationary growth phase in THY. For example, nine proteins of the histidine degradation pathway whose genes are located in a common chromosomal region (Spy49_1724 to Spy49_1731, as well as Spy49_1723c on the complementary strand) showed on average 22-fold higher abundances in the stationary and late stationary phase compared to the exponential phase. (Figure S3). The levels of the nine subunits of the V-type ATP synthase encoded by the contiguous genes Spy49_0129 to Spy49_0137 were similarly elevated (Figure S4). We hypothesize that the V-type ATPase is involved in pH regulation of cells grown in THY. Growth on fructose induced or enhanced the expression of several proteins, most strikingly of a group of nine proteins with less characterized functions encoded by the consecutive genes Spy49_0450 to Spy49_0460 (Figure S5). With regard to the phosphoproteomic analysis, it is important to point out that the expression profiles of these proteins indicate differences in the time course of metabolic adaptation to fructose utilization between experiments 1 and 2. In experiment 1, expression peaked already in the exponential growth phase (44-fold increase on average of the nine proteins compared to THYexp), while in experiment 2 the maximum expression was found in the stationary phase (135-fold increase on average compared to THYexp).

There were also various proteins whose abundance decreased in the stationary phase. These were often surface-exposed. Cultivation in CDM- did not allow growth of the bacterial cultures, as illustrated by the continuous decrease in optical density (Figure 1). Nevertheless, to standardize sample designations with the other culture conditions, we have also used the terms exp, stat, and late stat for the CDM- cultures in the tables and figures. Cultivation in CDM- caused lesser effects on the proteome, mainly a decrease in surface-exposed and membrane proteins with increasing cultivation duration. However, many ribosomal proteins, such as L28 and L35, were more reduced in CDM- than in the other media (Figure S6). Despite the considerable differences in the expression of many proteins, about 60% of the proteins remained almost unaffected (fold change < 2) by the culture conditions studied.

### 3.3. Identification of Phosphopeptides by Different Search Strategies

To study dynamic protein phosphorylation, we used label-free, data-independent LC-HDMS^E^ acquisition, which collects high-quality MS data across the entire chromatographic peak width [36]. Using Progenesis QI for proteomics for data analysis, this approach provides reliable quantification of unmodified peptides and proteins [34,41], but the proprietary ion accounting algorithm for peptide and protein identification was found to be error-prone in identifying phosphopeptides. Increasing the stringency of the ion accounting search enabled confident identification of the peptide sequences, but the location of the phosphorylation site was often ambiguous and required time-consuming manual validation. As an alternative to the ion accounting algorithm, peak lists were searched using the Mascot search engine. This strategy provided reliable phosphopeptide identifications supported by a Mascot Delta Score-based phosphosite localization probability [38]. Additionally, we used spectral libraries assembled from high-confidence MS/MS spectra of phosphopeptides obtained from the same experiments by data-dependent analysis (DDA) and Mascot search. Thus, four data sets were generated from each of the three experiments: (i) HDMS^E^ data searched with the ion accounting algorithm, (ii) HDMS^E^ data searched with Mascot, (iii) HDMS^E^ data searched against a spectral library, and (iv) DDA data searched with Mascot (Figure 1). For each experiment, the three identification results of the HDMS^E^ data were combined in a single Excel sheet, whereas the DDA results are shown separately (Table S2). Identification of the same feature using ion accounting, Mascot, or a spectral library search always revealed the same peptide sequence, but sometimes there were differences in phosphorylation site localization, which required further critical review.

**Figure 2:**
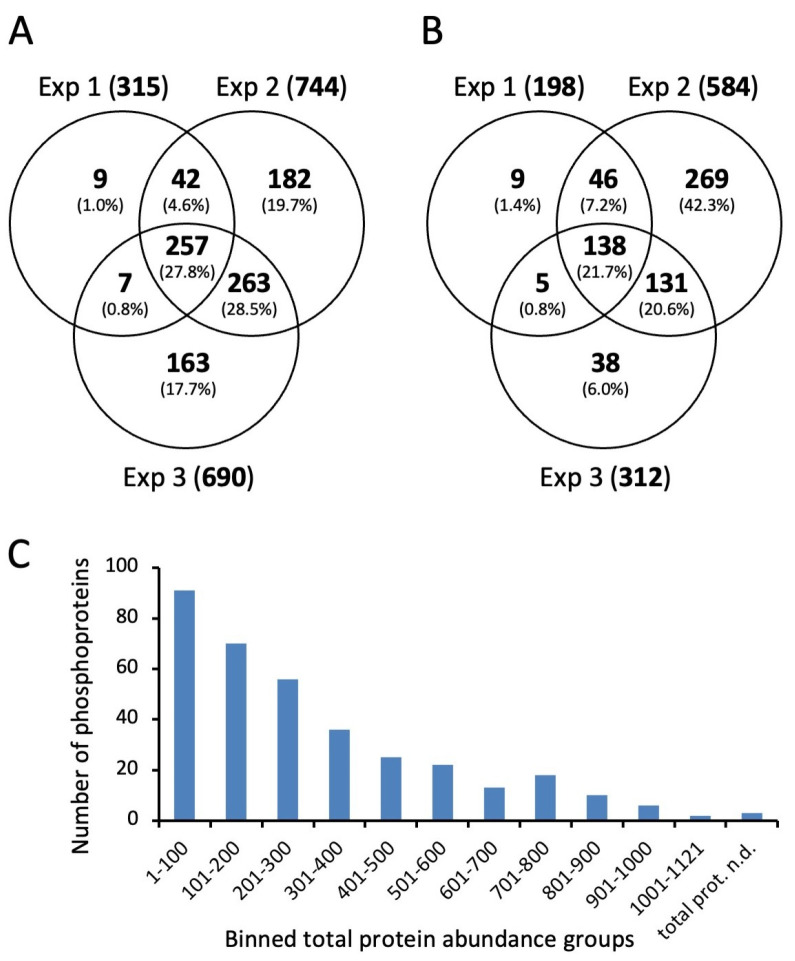
Identification of phosphorylated peptides without considering the phosphorylation site. (**A**) Venn diagram showing numbers and percentages of phosphorylated peptides identified in three experiments after HDMS^E^ acquisition; (**B**) Venn diagram showing numbers and percentages of phosphorylated peptides identified in three experiments after DDA acquisition; (**C**) dependency of phosphopeptide identification on protein abundance. Each bar represents 100 proteins ordered according to decreasing abundance.

In this study, we first considered the phosphopeptides without taking the phosphorylation site into account (Table 1). Most phosphorylated peptides were found in the second experiment, which included the largest number of different growth conditions. In all of the experiments, a total of 352 phosphoproteins derived from 955 peptide sequences were identified. The concordance between the identification of phosphorylated peptide sequences in the three experiments is shown in Figure 2A,B for the HDMS^E^ and DDA measurements, respectively. Almost all of the peptides identified in experiment 1 were also found in experiments 2 and 3, which additionally included samples from the late stationary phase. This indicates that the number of phosphorylation sites increased during the prolonged stationary phase.

There was a strong correlation between the protein abundance determined in proteomic analysis and the identification of phosphopeptides (Figure 2C). We identified phosphorylated forms of 91 of the 100 most abundant proteins. Of the nine nonphosphorylated proteins, three were surface-exposed proteins, such as metal ABC transporter substrate-binding lipoprotein MtsA and foldase protein PrsA, which would not be expected to be phosphorylated by cytoplasmic kinases. In the phosphoproteome of *S. pyogenes* M1, the majority of the identified phosphosites also originated from abundant proteins [26].

To determine which phosphoproteins dominate quantitatively, the phosphopeptide abundances of the individual proteins were summed for the exponential growth phase and the late stationary phase, respectively (Figures S7 and S8).

An evaluation of the phosphopeptide enrichment method revealed a high efficiency, as about 25% of the identified peptides were phosphorylated at serine, threonine, or tyrosine residues. In addition, an in-depth analysis of unidentified peptide ions revealed putative glycosylation of membrane-bound foldase PrsA and frequently occurring lysine modification by phosphoglycerylation [42,43] (Figures S9 and S10).

### 3.4. Creating a List of Phosphorylation Sites

To generate a list of all phosphorylation sites that were reliably identified by the HDMS^E^ and DDA approaches, all phosphopeptides with unambiguous site localization were merged and aligned to 15 amino acid stretches with a central phosphorylation site. Only phosphorylation sites identified in at least two conditions were included in the final list, e.g., from the same peptide ion identified in different experiments or with different search strategies, or from different peptide ions containing the same phosphorylation site. Using these stringent criteria, a list of 815 phosphorylation sites was created (Table S3). There were 240 sites (29.4%) identified in all three experiments, and 592 sites (72.6%) were identified in at least two experiments despite the different designs of the three experiments (Figure S11A). As expected, the different data acquisition and search strategies complemented each other. However, most phosphorylation sites (78.5%) were identified by searching the HDMS^E^ data using Mascot (Figure S11B).

The proportions of phosphorylated serine, threonine, and tyrosine residues were 73%, 21%, and 6%, respectively. The 815 phophorylation sites were distributed among 294 proteins. Of these, 10 proteins contained at least 10 phosphorylation sites; first among these was elongation factor Tu (EF-Tu) with 20 sites. On the other hand, only a single phosphorylation site was found in 131 proteins (Table S4). With few exceptions, the identified phosphopeptides were phosphorylated at one single site. However, we observed many positional isomers of singly phosphorylated peptides with reproducible differences in chromatographic retention time, as exemplified for five phosphorylation sites on the same tryptic peptide of the general stress protein Spy49_1001c (Figure S12).

### 3.5. Quantitative Analysis of Dynamic Protein Phosphorylation

**Figure 3:**
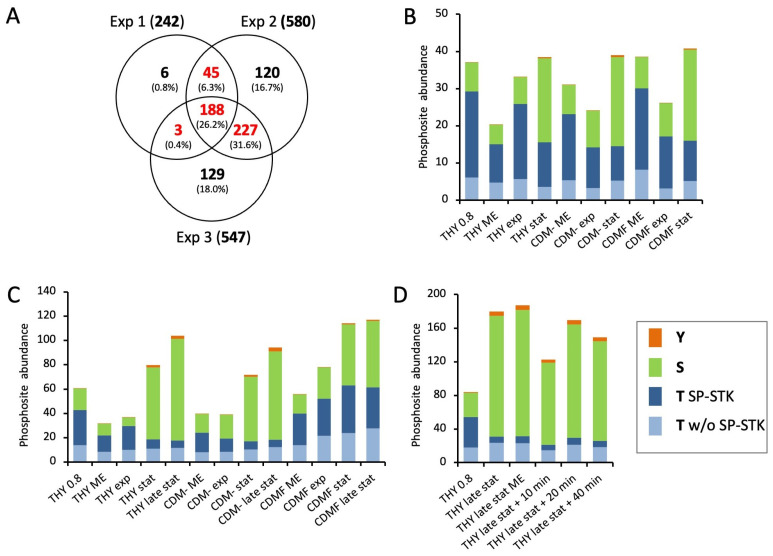
Quantitative analysis of dynamic protein phosphorylation. (**A**) Venn diagram showing numbers and percentages of phosphorylation sites quantified in HDMS^E^ analyses in three experiments. Colored numbers indicate phosphosites that were quantified in at least two experiments and included in the analysis. (**B**–**D**) Quantitative distribution of phosphorylation events at serine, threonine, and tyrosine in *S. pyogenes* cultures at different growth phases in different culture media in the first (**B**), second (**C**), and third (**D**) experiments. The heights of the bars indicate total phosphorylation. The proportions of threonine, serine, and tyrosine sites are color-coded. Threonine phosphorylation of the PASTA kinase SP-STK (T SP-STK) is shown separately from all other threonine phosphorylations (T w/o SP-STK).

For the quantitative analysis of dynamic protein phosphorylation, a subset of 463 phosphorylation sites quantified in HDMS^E^ analyses from at least two of the three experiments was considered (Figure 3A). For each phosphorylation site, the amounts of all associated phosphopeptide ions were summarized, i.e., species with different charge states, missed cleavages, and additional modifications (Table S5).

First, we analyzed the general dynamics of phosphorylation events at serine, threonine, and tyrosine during the course of the experiments. As a result of its dominance, threonine phosphorylation of SP-STK was quantified separately (Figure 3). In the first experiment, threonine phosphorylation was halved from almost 80% of the total phosphopeptide amount to about 40% in the stationary phase. At the same time, the proportion of serine phosphorylation increased from 20% to 60%. This trend was similar for all three growth media (Figure 3B). In the second experiment, the cultivation of the bacteria was extended to 72 h. During cultivation in THY and CDM-, threonine phosphorylation decreased even more than in the first experiment to about one-third in the stationary phase after 24 h and one-fourth in late stationary phase after 72 h, while the amount of serine phosphorylation and the total protein phosphorylation increased. In the exponential growth phase, 67% to 79% of threonine phosphorylation occurred at SP-STK. In particular, T324 of the kinase was by far the most frequent phosphorylation site. The decrease in total threonine phosphorylation in the stationary phase was mainly due to the reduced phosphorylation of SP-STK. Interestingly, the absolute amount of serine phosphorylation continued to increase after the bacteria entered the stationary phase or decline phase in THY and CDM-. Tyrosine phosphorylation also increased in the stationary and decline phases but accounted for only a small quantitative fraction of total phosphorylation. In CDMF, the changes in the ratio between threonine and serine phosphorylation were less pronounced in the second experiment (Figure 3C). This may be related to the differences in the time course of metabolic adaptation to fructose utilization observed in the analysis of the proteome (Figure S5). The third experiment confirmed the reversal of the ratio of threonine and serine phosphorylation between the exponential growth phase and the late stationary phase in THY. Transfer of cultures from late stationary phase to fresh medium had no significant effect on phosphopeptide abundance during a 40-minute period (Figure 3D). It should be noted that results of single phosphopeptide enrichents from single bacterial cultures are shown, yielding a large margin of error for individual time points (Figure 3). However, the described main trends were clearly reproduced by the experiments.

**Figure 4:**
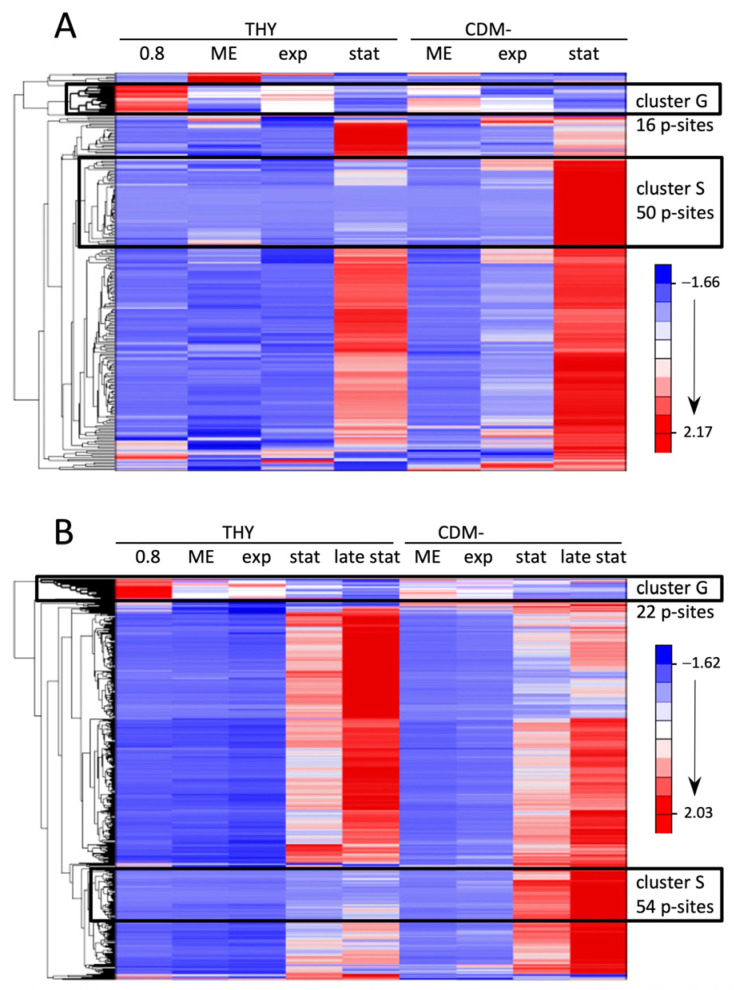
Hierarchical clustering of protein level-normalized phosphorylation site abundances in *S. pyogenes* cultures at different growth phases in THY and CDM-. (**A**) Quantitative data of 236 phosphosites from the first experiment (color coded in Figure 3A) are included. (**B**) Quantitative data of 460 phosphosites from the second experiment (color coded in Figure 3A) are included. The G and S clusters are outlined and the number of phosphorylation sites in each is indicated.

Next, we analyzed the individual phosphorylation events during the course of the growth experiments. To exclude the influence of altered protein expression, the phosphosite values were normalized to the corresponding protein levels. Hierarchical clustering was performed to visualize groups of differentially phosphorylated sites (Figure 4 and Figure S1B). Cultures grown in CDMF are not included due to their inconsistent growth characteristics described above, but cluster analyses of all growth conditions including CDMF are provided as Supplementary Material (Supplementary Data Sheet S1). In each of the three experiments, there was a clearly defined cluster containing sites whose phosphorylation level was highest during exponential growth and significantly reduced in the stationary or decline phase (designated as cluster G for growing cells, Figure 4 and Figure S1B). These clusters contained largely consistent phosphorylation sites, 20 of which are listed in Table 2. It is noteworthy that 17 of the 20 phosphorylations occurred at threonine residues, indicating that threonine phosphorylation is strongly overrepresented in this group.

Most of the phosphorylation sites, including four phosphosites of SP-STK, belong to proteins involved in the cell cycle of Gram-positive bacteria [54]. Several of the sites have been previously characterized as targets of PASTA kinases in related bacteria, such as T201 and T245 of the cell division initiation protein DivIVA, T66 and T86 of the cell cycle protein GpsB, T7 of the cell division protein FtsZ, T30 of the MacP ortholog Spy49_0377 [51], and T7 of Spy49_1751c, which belongs to a group of highly conserved cell cycle-related proteins in low-GC Gram-positive bacteria [52,53] (Table 2). PASTA kinase-dependent phosphorylation of mid-cell-anchored protein Z (MapZ/LocZ) [55,56] and endolytic murein transglycosylase (MltG) was also detected in other bacteria, but at different sites than in our analysis. Other peptides of cell cycle-related proteins whose phosphorylation sites could not be localized with certainty also exhibited phosphorylation profiles comparable to the phosphosites in cluster G. These phosphopeptides derived from the cell division proteins SepF (putative p-site T121), FtsA (putative p-site S416 or T417), FtsZ (putative p-site T336), and MapZ (putative p-site T73), and from MltG (putative p-site T89) and MacP ortholog Spy49_0377 (putative p-sites T7 and T19) (Table S2). Thus, our investigation of phosphorylation dynamics during certain growth phases enabled the identification of specific phosphorylation events and phosphopeptides with possible functions in the cell cycle of *S. pyogenes*.

**Figure 5:**
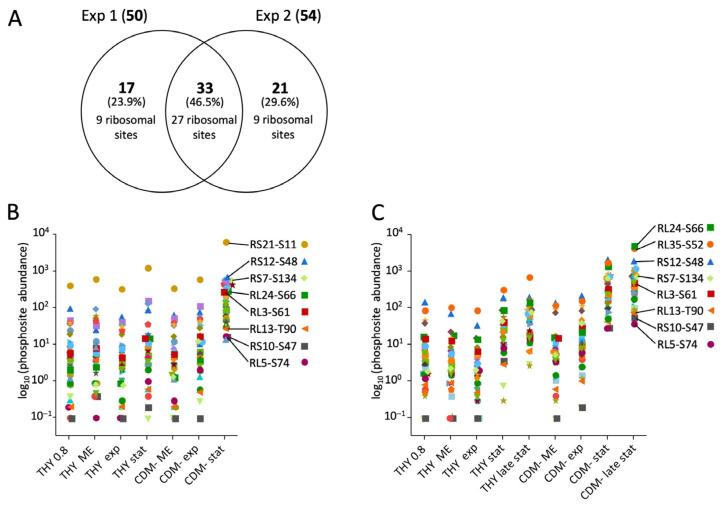
Increased phosphorylation of ribosomal proteins during starvation. (**A**) Venn diagram showing the number and percentage of phosphorylation sites and the number of ribosomal sites in the S cluster of the first and second experiments (see Figure 3); (**B**) culture condition-dependent abundance of 36 ribosomal phosphorylation sites included in the S cluster of the first experiment; (**C**) culture condition-dependent abundance of 36 ribosomal phosphorylation sites included in the S cluster of the second experiment. Phosphorylation site values were normalized to the corresponding protein levels. Selected phosphorylation sites were labeled with the protein short description and, separated by a hyphen, with the phosphorylation site.

Many phosphosites showed detectable phosphorylation only in the stationary phase or during starvation. Even in starving bacteria in CDM-, whose optical density decreased throughout the experiment, phosphorylation increased during the prolonged cultivation of 72 h. The increase was absolute and not just a result of normalization to decreasing protein levels. A particularly striking increase in phosphorylation in CDM- was characteristic of two clusters comprising 50 and 54 phosphorylation sites in experiments 1 and 2, respectively (designated as cluster S for starving cells in Figure 4). Together they contained 71 different phosphosites, of which 33 were present in both clusters (Figure 5A). A total of 27 of these common phosphosites originated from ribosomal proteins. Additionally, there was a high proportion of ribosomal phosphorylation sites among the phosphosites unique to each cluster. Thus, of the 50 phosphosites forming cluster S from experiment 1, 36 originated from ribosomal proteins, which corresponds to 84% of all ribosomal phosphosites found in experiment 1. Similarly, 36 ribosomal phosphosites, corresponding to 53% of all ribosomal phosphosites found in experiment 2, were enriched in cluster S of experiment 2 (Figure 5A). Compared to the exponential growth phase in THY, phosphorylation of these ribosomal sites increased by means of 301-fold and 507-fold in the stationary and late stationary phases in CDM-, respectively (Figure 5B,C).

**Figure 6:**
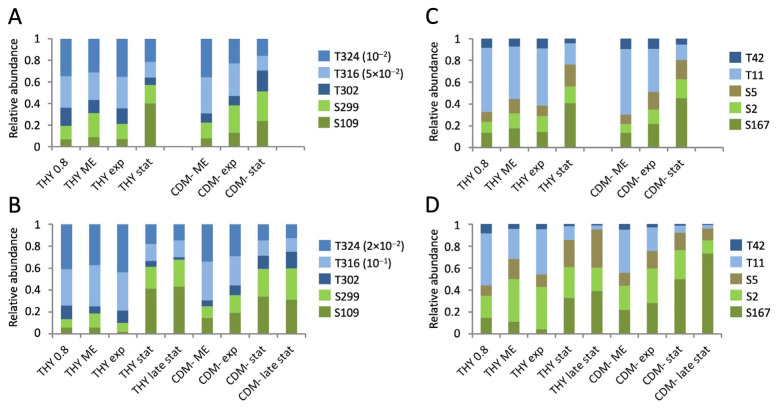
Growth phase-dependent phosphorylation of threonine and serine residues located on the same peptide or protein. (**A**,**B**) Phosphorylation dynamics of SP-STK in the first (**A**) and second (**B**) experiments. T302 and S299 are located on the same tryptic peptide of SP-STK. (**C**,**D**) Phosphorylation dynamics of MapZ in the first (**C**) and second (**D**) experiments.

Next, we investigated opposing dynamics of phosphorylation events on serine and threonine residues on the same peptide or protein, as shown in Figure 6 for SP-STK and mid-cell-anchored protein Z. The phosphorylated residues T302 and S299 of SP-STK are located on the same tryptic peptide. During the stationary phase in THY, the phosphorylation of T302 decreased in parallel to the phosphorylation intensity of the very abundant phosphosites T324 and T316, while the phosphorylation of S299 slightly increased. However, in CDM-, the phosphorylation intensity of T302 did not decrease, indicating nutrient-dependent differences in the dynamics of phosphorylation (Figure 6A,B). The mid-cell-anchored protein Z showed a strong decrease in phosphorylation intensities of T11 and T42 with a concomitant increase in phosphorylation of S2, S5, and S167 during prolonged stationary phase in THY and CDM- (Figure 6C,D).

Opposite phosphorylation dynamics of nearby threonine and serine residues located on the same tryptic peptide were also exhibited by the cell cycle protein GpsB (T86/S84), the cell division protein FtsZ (T7/S4), and the protein translocase subunit SecA (T809/S806) (Figure S13).

Finally, we searched for phosphosites that were specifically regulated during growth with fructose. Despite the differences in adaptation to fructose utilization described above, we found reproducible phosphorylation events in experiments 1 and 2, such as significantly increased phosphorylation of T155 of ribonucleoside diphosphate reductase and of S13 of uridylate kinase, proteins involved in deoxyribonucleotide biosynthesis and pyrimidine metabolism, respectively (Figure S14). While the relationship of these proteins to fructose utilization is not obvious, proteins of the carbohydrate metabolism also showed changed phosphorylation patterns. Notably, enolase phosphorylation sites S2 and S42 were oppositely phosphorylated, with residue S2 being most phosphorylated in THY and CDM-, and residue S42 being most phosphorylated in CDMF (Figure S15).

## 4. Discussion

### 4.1. Protein Phosphorylation in S. pyogenes M49

We investigated S/T/Y phosphorylation events in *S. pyogenes* M49 at different growth phases in three culture media and identified 955 phosphorylated peptides derived from 352 proteins, out of which we extracted 815 high-confidence phosphorylation sites from 294 proteins. There were 162 identical phosphorylation sites found in the phosphoproteome of *S. pyogenes* M1 [26]. Here, threonine was preferentially phosphorylated, accounting for 55% of the phosphorylation sites. Different culture conditions may be responsible for this remarkable difference from our results, in which 73% of the phosphorylation events occur at serine.

The quantitative analysis of dynamic phosphorylation, which included a subset of 463 phosphorylation sites for which HDMS^E^ data were available from at least two of the three experiments, revealed the following trends: (i) the sum of Ser/Thr/Tyr phosphorylations increased in the stationary phase; (ii) the total amount and the relative proportion of threonine phosphorylation decreased in the stationary phase, primarily due to the decrease in threonine phosphorylation of SP-STK; (iii) the total amount and the relative proportion of serine phosphorylation increased in the stationary phase; and (iv) tyrosine phosphorylation behaved similarly to serine phosphorylation, but its proportion was low (max. 3.4%). Starved bacteria in CDM- exhibited similar phosphorylation patterns as cultures in the stationary phase.

### 4.2. Specific Threonine Residues of Cell Cycle-Related Proteins Are Putative Targets of the PASTA Kinase SP-STK during the Exponential Growth Phase

Hierarchical clustering of phosphosites normalized to protein abundance revealed a clearly differentiated cluster of decreasing phosphorylation events in the stationary phase, with largely consistent phosphosites in each of the three experiments. This cluster contains several abundant pT sites including four phosphorylated threonine residues within the juxtamembrane region of SP-STK, of which T324 displayed the highest signal of all identified phosphopeptides. Phosphorylated threonine residues in the juxtamembrane region have been reported in *S. pneumoniae* [44] and other bacteria, and are proposed to contribute to the activation of PASTA kinases and/or the docking of their targets [11]. PASTA kinases autophosphorylate threonine residues of their activation loop in response to specific stimuli [11]. The corresponding phosphosites were not found, perhaps due to their location on a large peptide of 34 amino acids in length, which is in the upper size range of identifiable peptides in our analyses.

Cluster analysis revealed 16 phosphosites in 12 proteins with phosphorylation dynamics comparable to the pT sites in SP-STK. Many of these proteins belong to the divisome, a protein complex assembled during cell division [57], and are known to be substrates of the PASTA kinase during regulation of cell division and morphogenesis in *S. pneumoniae* and other Gram-positive bacteria [54]. Corresponding phosphorylation patterns of the kinase IreK and its substrate IreB following growth signals or cell wall stress were also found in *Enterococcus faecalis* [58]. Since several of the 16 phosphosites are targets of PASTA kinases in related bacteria (Table 2), it can be assumed that they are phosphorylated by *S. pyogenes* SP-STK and play a functional role in the cell cycle. Selected proteins are discussed below.

GpsB is a DivIVA-like-domain-containing protein present only in *Firmicutes* [59]. In *S. pneumoniae*, GpsB is involved in the coordination of septal and peripheral peptidoglycan synthesis and in the regulation of the levels of StkP-mediated protein phosphorylation [57,60,61]. GpsB proteins comprise conserved N-terminal and C-terminal domains connected by a poorly conserved linker. The N-terminal domain interacts with the cytoplasmatic membrane [62] and with penicillin-binding proteins [63], while the C-terminal domain provides hexamerization of GpsB [64]. We identified growth phase-dependent phosphorylation of GpsB at T66 and T86. Both sites were also phosphorylated by the PASTA kinase in *Streptococcus suis* [45,46]. Whereas T66 is located within the less conserved linker region of GpsB, T86 is the first amino acid of the conserved C-terminal domain (Figure S16). In *E. faecalis*, the orthologous residue T84 was also a substrate of the PASTA kinase IreK [50]. In *S. pneumoniae*, T79, located in the aligned sequences four residues upstream of T86 of *S. pyogenes*, was found to be phosphorylated by StkP [44]. The *B. subtilis* kinase PrkC phosphorylated GpsB in vitro at T75, which is located one residue upstream of the conserved threonine residue in the aligned sequences. Unphosphorylated GpsB stimulated autophosphorylation of PrkC, whereas phosphorylation of GpsB reduced the kinase activity, thus providing a negative feedback loop [65]. Although the function of GpsB differs between bacterial species and strains [59,61], PASTA kinase-dependent phosphorylation at or near the conserved threonine residue at the beginning of the C-teminal domain appears to be conserved in *Firmicutes*, and may serve an important regulatory function in the cell cycle.

The uncharacterized protein Spy49_1748c was phosphorylated at T13. This multi-pass membrane protein is a member of the competence-induced protein Ccs4 protein family (IPR016978). A role of PASTA kinases in the regulation of competence induction, which is also related to the cell cycle, has been demonstrated for various streptococcal species [66,67,68] and could also exist in *S. pyogenes*.

The secretion motor ATPase SecA was phosphorylated at T809. SecA is a potential target of kinases in *Staphylococcus aureus* [9], *S. suis* [46], and *Clostridioides difficile* [10]. Most interesting is that *B. subtilis* SecA is required for membrane targeting of DivIVA [69], and in *L. monocytogenes* an interaction between DivIVA and SecA2 has been described too [70]. Therefore, it is possible that phosphorylation of SecA at T809 exerts a function in the cell cycle in *S. pyogenes*. Together, our results suggest that the PASTA kinase-dependent cell cycle regulatory processes found in related bacteria are also conserved in *S. pyogenes*.

The above-discussed phosphorylation events within the juxtamembrane region of SP-STK and at nine potentially cell cycle-related proteins (Table 2) occurred exclusively at threonine residues. A strong enrichment of phosphothreonine among peptides phosphorylated under the control of PASTA kinases and dephosphorylated under the control of their cognate phosphatases was recently reported for *C. difficile* [10] and is obvious from published lists of PASTA kinase substrates of various bacteria [9,44,45,46,47,48,49,50]. Thus, either the substrate specificities of these kinases are generally biased towards threonine, or the regulation of cell cycle events by the PASTA kinases occurs predominantly at threonine, while other functions of the kinase may also target serine residues.

### 4.3. Mostly Serine Residues Are Increasingly Phosphorylated during Stationary Phase and Starvation

Many of the cell cycle-related proteins that were phosphorylated at threonine residues during growth were phosphorylated at other, mostly serine residues in the stationary phase and during starvation. Some of these opposing phosphorylation events occurred in close proximity on the same peptide. Similar growth phase-dependent phosphorylation of the cell division proteins DivIVA and SepF at threonine and serine residues was found in *C. difficile* [71]. In our analysis, SP-STK and its cognate protein phosphatase SP-STP were also increasingly phosphorylated at multiple serine residues during the stationary growth phase. It would be attractive to speculate that these phosphorylation events may be specific on–off switches for cell cycle regulation. However, since a large number of proteins with a variety of cellular functions exhibit strongly increased serine phosphorylation in the stationary phase, a specific function of these phosphorylation events for the cell cycle proteins seems unlikely.

Recently, a sharp increase in the number of phosphopeptides after the onset of the stationary growth phase was observed in *C. difficile* [71]. Already, earlier studies found global increases in protein phosphorylation levels in later phases of growth in *E. coli* [72] and *B. subtilis* [73]. Therefore, the increase in protein phosphorylation after the onset of the stationary growth phase seems to be a general feature in the physiology of bacteria. Accordingly, the doubling of the number of phosphorylation sites in *S. pyogenes* in our research compared with a recently published phosphoproteome of the M1 serotype [26] is probably due to the inclusion of the prolonged stationary phase in our analysis.

In accordance with our findings in *S. pyogenes*, components of the translational machinery were increasingly phosphorylated during the stationary growth phase in *E. coli* and *B. subtilis* [72,73]. Phosphorylation of ribosomal proteins influences subunit association, binding sites, and translational activity of the ribosomes [74,75]. Phosphorylated residues were located (mostly solvent accessible) on the surface of ribosomal proteins [74]. In *E. coli*, phosphorylation of bL9 was found to be important for cell survival under starvation stress [76].

We identified the largest number of phosphosites within a single protein in EF-Tu. Of the total 20 phosphosites, 14 could be used for the quantitative analysis, all of which increased in abundance during the stationary growth phase. Hyperphosphorylation of EF-Tu in the stationary phase has been observed in studies with various bacteria. For example, 32 phosphosites were recently identified in *C. difficile* [71]. Phosphorylation of *E. coli* EF-Tu at T382, which was first reported in 1993 [77], and at other residues, has been shown to inhibit protein synthesis in Gram-negative and Gram-positive bacteria [17,78,79,80]. Among several models for the function of EF-Tu phosphorylation in inhibiting protein synthesis, one describes phosphorylation at *E. coli* T382 or at the equivalent site of other bacteria as a switch that interrupts the conformational cycle and traps EF-Tu in an open state. As a result, sites buried in the closed conformation become accessible to the solvent and can be phosphorylated, leading to hyperphosphorylation [80]. This model used phosphosites previously identified in *E. coli* [5]. Interestingly, we found that the corresponding phosphosites in *S. pyogenes* are increasingly phosphorylated in the stationary phase and during starvation. This may indicate their function in regulating translation. On the other hand, the phosphorylation events at the stationary phase occurred at substoichiometric levels, as can be deduced from the abundance profiles of the corresponding unphosphorylated peptides. This questions their physiological relevance. A new interesting aspect is the recently described role of phosphoregulation for the bacterial quiescence [81]. The possibility that the reduced protein turnover in the stationary phase could lead to an accumulation of non-specific phosphorylations should also be considered. Hence, further research is needed to elucidate the function(s) of enhanced protein phosphorylation in the stationary phase and in starving cells. It is also not known which kinases are responsible for these phosphorylation events, and how they relate to the activity of phosphatases.

### 4.4. Most Proteins Can Probably Be Phosphorylated during Stationary Phase and Starvation—Is There an Unknown, Rather Non-Specific Kinase?

The massive increase in phosphorylation during the stationary phase was not limited to certain groups of proteins such as components of the translational machinery, e.g., several components of the phosphotransferase system were among the quantitatively predominant phosphoproteins (Figure S8). Our finding of phosphopeptides from 91 of the 100 most abundant proteins indicates a strong correlation between protein abundance and the identifiability of phosphopeptides. It can be assumed that, with the exception of certain proteins whose localization or specific structure prevents their phosphorylation, the vast majority of proteins can be phosphorylated. However, identification of their phosphopeptides will depend on the sensitivity of the proteomic methods used. As previously suspected for different bacteria [9,10,81], other non-Hanks-type kinases besides PASTA kinase must be involved to phosphorylate the broad substrate spectrum in *S. pyogenes*.

In contrast to many bacteria including *S. pneumoniae*, *S. pyogenes* has no bacterial-type tyrosine kinase (BY-kinase) [14]. However, it possesses a low-molecular-weight protein tyrosine phosphatase [82] and a member of the recently described class of ubiquitous bacterial kinases (Ubk) [16]. In vitro, recombinant UbK of *S. pyogenes* strain M1T1 5448 autophosphorylated on two tyrosine residues and phosphorylated the response regulators CovR and WalR, the Ser/Thr phosphatase SP-STP, and GAPDH, each at several serine and/or threonine and/or tyrosine residues [83]. Interestingly, we found several corresponding phosphorylation sites on CsrR (CovR ortholog), SP-STP, and GAPDH. Of note, recombinant SP-STP and GAPDH used for in vitro phosphorylation were slightly phosphorylated already during production in *E. coli* [83], indicating that phosphorylation of the respective sites does not require a very specific kinase. Therefore, UbK is an interesting candidate, but the extent to which the kinase contributes to phosphorylation in *S. pyogenes* requires further experiments. With the progress in automated annotation, two previously uncharacterized proteins (Spy49_0994c and Spy49_0456) were recently annotated in the TrEMBL database as serine/threonine protein kinases. Their functional characterization is still pending, and they should be considered in future studies on protein phosphorylation in *S. pyogenes*.

## 5. Conclusions

The quantitative analysis of dynamic protein phosphorylation in *S. pyogenes* during growth, stationary phase, and starvation revealed two main types of phosphorylation events, distinguished by the growth phase in which they predominantly occur and their preference for either threonine or serine. One small group of phosphorylation events occurred nearly exclusively on threonine residues of cell cycle-related proteins and was enhanced in growing cells. Data from the literature support the assumption that these are targets of the PASTA kinase SP-STK. The majority of phosphorylation events occurred in the stationary phase or in starving bacteria. Their function and the kinases responsible for their formation need to be elucidated in further studies. Since our research has shown that the growth phase has a decisive influence on the phosphoproteome, and therefore on the activity of kinases and possibly phosphatases, future studies of kinase targets using mutants should include several different growth phases.

## Figures and Tables

**Table 1:** Number of phosphopeptide features, phosphorylated peptides without taking into account the phosphorylation site, and phosphoproteins identified in three independent experiments.

	Experiment 1 24 h, Three Media		Experiment 2 72 h, Three Media		Experiment 3 72 h, Only THY	
	HDMS^E^	DDA	HDMS^E^	DDA	HDMS^E^	DDA
Features	608	278	1419	905	1380	522
Peptides	315	198	744	584	690	312
Proteins	154	106	300	258	279	168

**Table 2:** Phosphosites whose phosphorylation level was highest during growth and decreased in the stationary phase and during starvation.

UniProt Accession	UniProt Protein Name	p-Site	Exp 1	Exp 2	Exp 3	Birk et al., 2021 [26] ^1^	Reference ^2^
A0A0H3BY55	Non-specific serine/threonine protein kinase	T302	+ ^3^	+	+	−	
A0A0H3BY55	Non-specific serine/threonine protein kinase	T316	+	+	+	+	
A0A0H3BY55	Non-specific serine/threonine protein kinase	T324	+	+	+	+	
A0A0H3BY55	Non-specific serine/threonine protein kinase	T291	n.i.	+	+	+	[44]
A0A0H3BZ18	Cell division initiation protein	T201	+ ^3^	+	+	+	[44,45,46,47,48,49]
A0A0H3BZ18	Cell division initiation protein	T245	+ ^4^	+	+	+	[48]
B5XMJ7	Cell cycle protein GpsB	T66	+	+	+	+	[46]
B5XMJ7	Cell cycle protein GpsB	T86	+	+	+	+	[46,50]
A0A0H3BZR7	Mid-cell-anchored protein Z	T11	+	+	+	+	
A0A0H3BZR7	Mid-cell-anchored protein Z	T42	+	+	+	+	
A0A0H3BZ23	Cell division protein FtsZ	T7	+	+	n.i.	−	[44,49]
A0A0H3BZC1	Uncharacterized protein Spy49_0377	T30	+	+	+	+	[49,51]
B5XJ02	UPF0297 protein Spy49_1751c	T7	+ ^3^	+	+	+	[9,44,45,46,49,50,52,53]
B5XI23	Protein translocase subunit SecA	T809	+	+	+	+	
A0A0H3BXH0	Endolytic murein transglycosylase	T122	+ ^3^	+	n.i.	+	
A0A0H3C2P8	Uncharacterized protein Spy49_1748c	T13	+	+	+	+	
A0A0H3C0J7	Phosphocarrier protein HPr	S31	−	+	+	−	[46]
A0A0H3C2S7	Uncharacterized protein Spy49_1801c	S148	+	+	n.i.	+	
A0A0H3C2S7	Uncharacterized protein Spy49_1801c	T125	+	+	n.i.	+	
A0A0H3BZP2	Arsenate reductase	S131	+ ^4^	+	+	+	

^1^ + indicates phosphosites, which were also identified in the phosphoproteome of *S. pyogenes* M1 [26]. ^2^ References are given for sites identified by mutant analysis as targets of PASTA kinases in other bacteria: *Streptococcus pneumoniae* [44,47,51]; *Streptococcus thermophilus* [48]; *Streptococcus suis* [45,46]; *Streptococcus agalactiae* [49]; *Enterococcus faecalis* [50,52]; *Staphylococcus aureus* [9]; *Listeria monocytogenes* [53]. ^3^ Phosphosite was not included in cluster G of the first experiment, but was located directly next to it. ^4^ Phosphosite clustered separately in the first experiment, although the phosphorylation level decreased at stationary phase. n.i., not identified.

## Data Availability

The mass spectrometry proteomics data have been deposited to the ProteomeXchange Consortium via the PRIDE [39] partner repository with the dataset identifier PXD044423 and 10.6019/PXD044423.

## Supplementary Materials

The following supporting information can be downloaded at: https://www.mdpi.com/article/10.3390/microorganisms12030621/s1, Figure S1: Bacterial growth and hierarchical clustering of protein level-normalized phosphorylation site abundances of the third experiment; Figure S2: Venn diagrams comparing proteins identified in three experiments; Figure S3: Expression profiles of coordinately regulated proteins of the histidine degradation pathway; Figure S4: Expression profiles of the coordinately regulated V-type ATP synthase subunits; Figure S5: Expression profiles of a group of fructose-induced proteins; Figure S6: Expression profiles of selected ribosomal proteins; Figure S7: Quantitatively predominant phosphoproteins in the exponential growth phase; Figure S8: Quantitatively predominant phosphoproteins in the late stationary growth phase; Figure S9: Evaluation of the phosphopeptide enrichment method; Figure S10: Evidence that foldase protein PrsA (PrtM1) is a glycoprotein; Figure S11: Venn diagrams showing numbers and percentages of high-confidence phosphorylation sites identified in each of the three experiments; Figure S12: Detection of phosphopeptide positional isomers; Figure S13: Growth phase-dependent phosphorylation of nearby threonine and serine residues located on the same tryptic peptide; Figure S14: Increased phosphorylation events during cultivation in CDMF; Figure S15: Phosphorylation dynamics of enolase and isoleucine-tRNA ligase at different culture conditions; Figure S16: Protein sequence alignment of GpsB; Table S1: Label-free quantification of the proteome; Table S2: Detailed data on phosphopeptide identification; Table S3: List of 815 phosphorylation sites; Table S4: List of 294 phosphoproteins; Table S5: Quantification of phosphorylation sites; Data Sheet S1: Cluster analyses of all growth conditions including CDMF.

## Abbreviations

PASTA: penicillin-binding protein and serine/threonine-associated; SP-STK: *S. pyogenes*-serine/threonine kinase; SP-STP: *S. pyogenes*-serine/threonine phosphatase; Ubk: ubiquitous bacterial kinase; THY: Todd-Hewitt broth supplemented with yeast extract; CDM-: chemically defined medium without carbon source; CDMF: chemically defined medium with fructose; HDMS^E^: data-independent acquisition mode with ion mobility separation as an additional dimension of separation; DDA: data-dependent acquisition.
