# Supplementary material for: Dynamic Protein Phosphorylation in Streptococcus pyogenes during Growth, Stationary Phase, and Starvation
Source: Microorganisms. 2024 Mar 20;12(3):621. doi: 10.3390/microorganisms12030621 (PMC10975399; doi:10.3390/microorganisms12030621)
Supplement: Supplementary file 1 [file microorganisms-12-00621-s001.zip › Supplementary Figures S1-S16.pdf]

Supplementary Figures S1-S16

Dynamic protein phosphorylation in *Streptococcus pyogenes* during growth, stationary phase, and starvation

Stefan Mikkat[1]*, Michael Kreutzer[2], Nadja Patenge[3]*

[1] Core Facility Proteome Analysis, Rostock University Medical Center, 18057 Rostock, Germany
[2] Medical Research Center, Rostock University Medical Center, 18057 Rostock, Germany
[3] Institute of Medical Microbiology, Virology and Hygiene, Rostock University Medical Center, 18057 Rostock, Germany

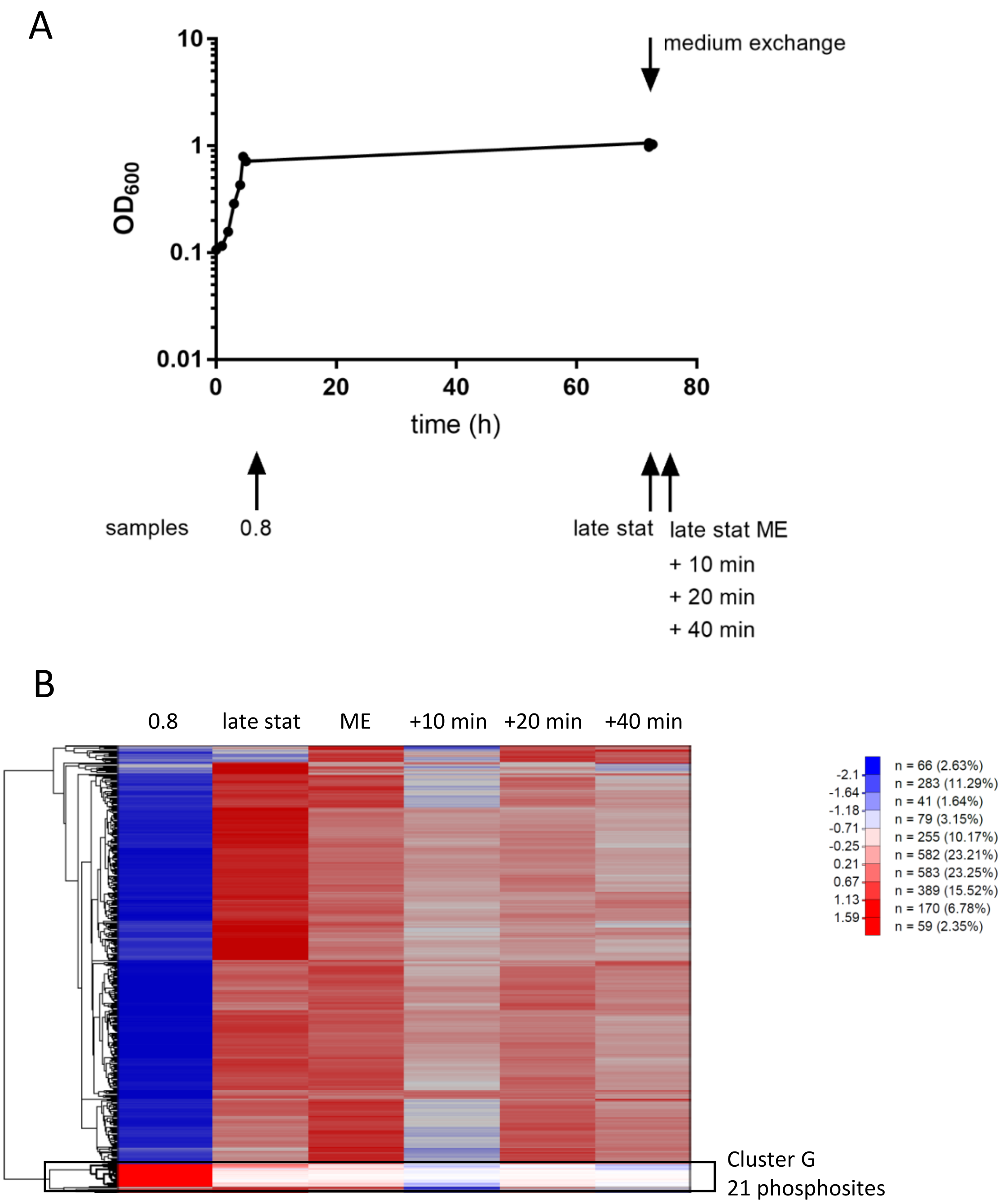

Figure S1. Bacterial growth and hierarchical clustering of protein level-normalized phosphorylation site abundances of the third experiment. **(A)** *S. pyogenes* was grown in THY for 72 h until the late stationary phase. For medium exchange (ME), bacterial cultures were centrifuged, pellets were suspended in fresh THY and incubated for 10, 20, and 40 min. Sample collection is indicated by arrows. 0.8: OD_{600} = 0.8; late stat: late stationary phase. **(B)** Hierarchical clustering of 418 protein level-normalized phosphorylation site abundances. The G cluster is outlined and the number of its phosphorylation sites is indicated.

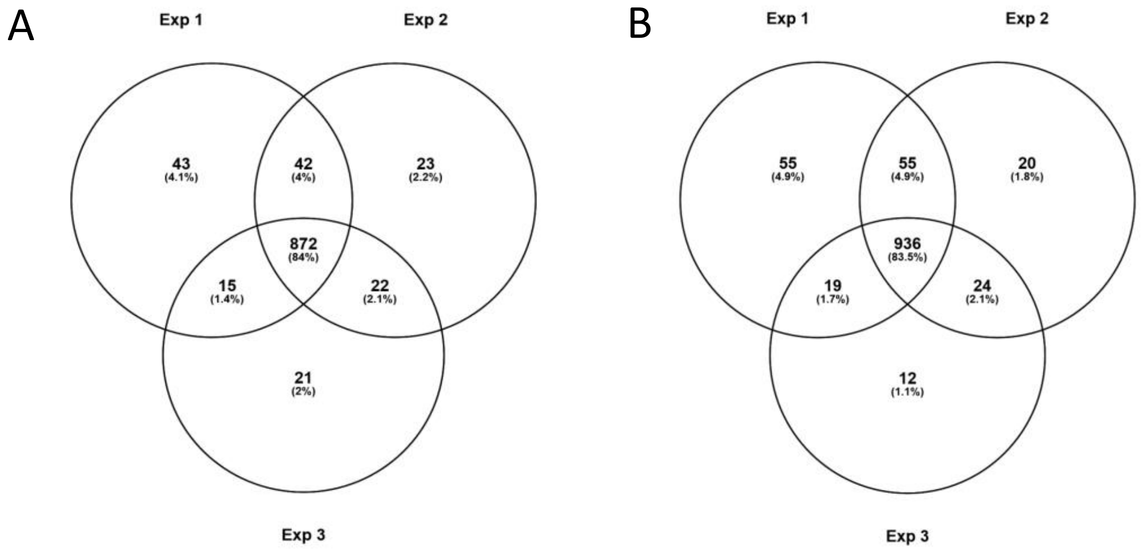

Figure S2. Venn diagrams comparing proteins identified in three experiments. **(A)** Proteins identified by at least two unique peptides. **(B)** Proteins identified by at least one unique peptide.

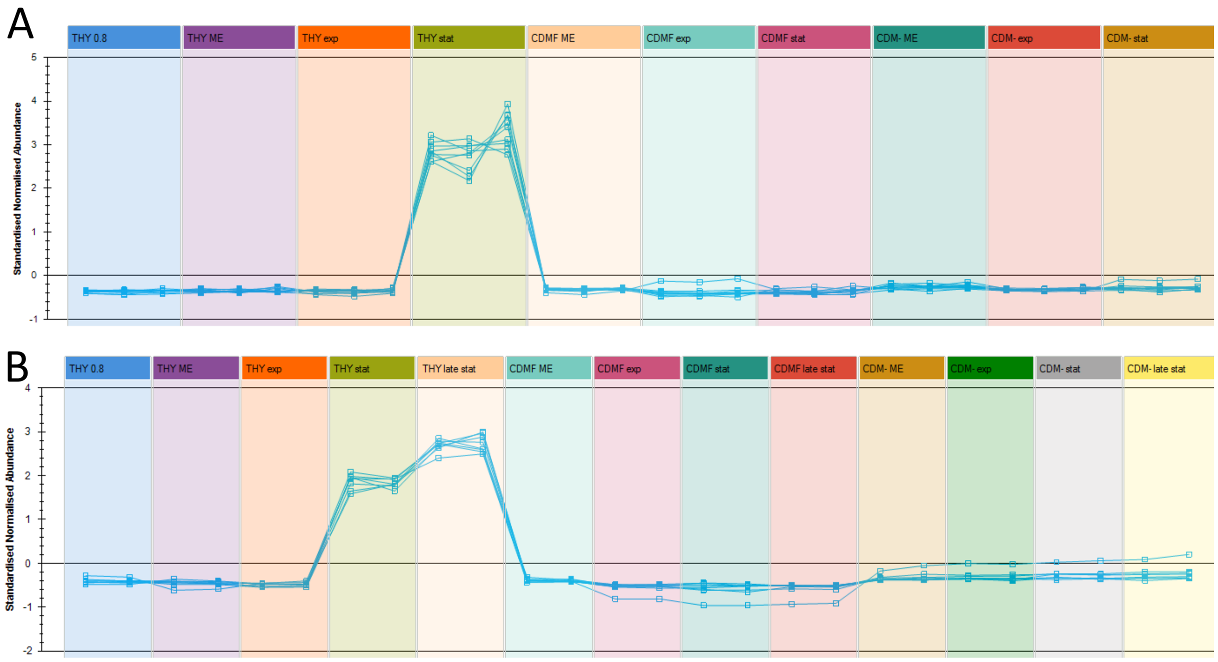

C

UniProt Acc.	Protein name	Gene	Max. FC[1]	
			Exp. 1	Exp. 2
B5XIX5	Imidazolonepropionase	*hutI* - Spy49_1723c	19.2	33.7
A0A0H3C083	Urocanate hydratase	*hutU* - Spy49_1724	33.7	15.5
A0A0H3C0S8	Glutamate formimidoyltransferase	Spy49_1725	27.5	52.1
A0A0H3C1X3	Formiminotetrahydrofolate cyclodeaminase	Spy49_1726	12.9	21.3
A0A0H3C2K8	Formate--tetrahydrofolate ligase	*fhs2* - Spy49_1727	19.8	79.4
A0A0H3BZ97	Conserved protein	Spy49_1728	79.7	n.d.[2]
A0A0H3C089	Putative cationic amino acid transporter	Spy49_1729	85.6	155.3
B5XIY2	Histidine ammonia-lyase	*hutH* - Spy49_1730	18.4	13.9
B5XIY3	Formimidoylglutamase	*hutG* - Spy49_1731	21.1	20.3

Figure S3. Expression profiles of coordinately regulated proteins of the histidine degradation pathway during cultivation in either THY medium (THY), CDM with fructose (CDMF) or CDM without a carbon source (CDM-). The proteins were significantly expressed only during stationary phase in THY. Results from experiment 1 **(A)** and experiment 2 **(B)** are shown. Protein identities and the maximal fold change are indicated in the table **(C)**.

[1)] maximal fold change is the highest abundance difference between two growth conditions of the experiment;
[2)] protein was not identified in this experiment.

A

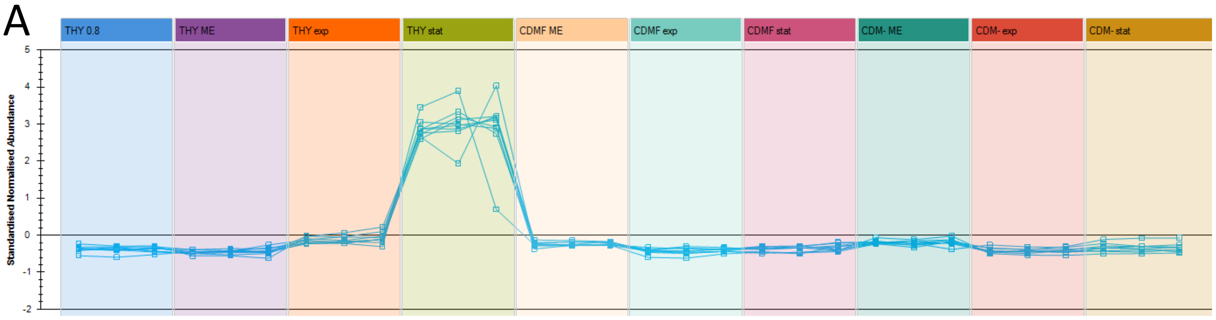

B

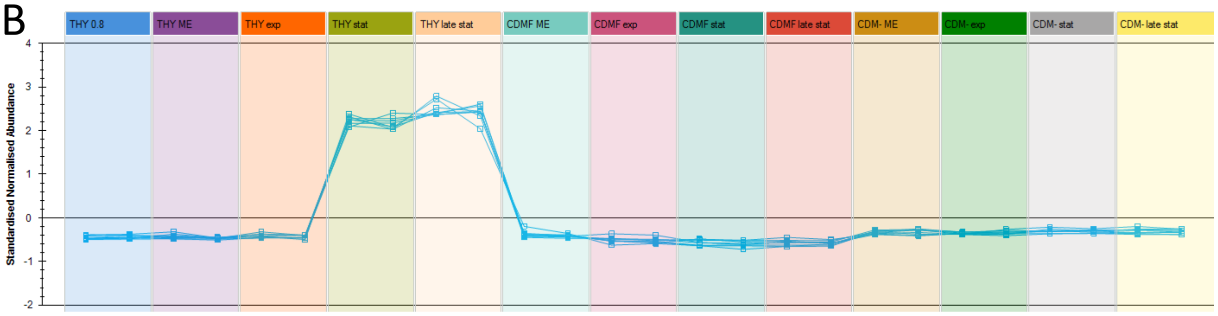

C

UniProt Acc.	Protein name	Gene	Max. FC[1]	
			Exp. 1	Exp. 2
A0A0H3BX58	Uncharacterized protein	Spy49_0129	6.7	32.4
A0A0H3BYG2	V-type ATP synthase subunit I	Spy49_0130	35.9	32.0
A0A0H3BYV4	V-type Na+-ATPase subunit K	Spy49_0131	7.6[2]	n.d.[3]
A0A0H3BVI6	Putative V-type Na+-ATPase subunit E	Spy49_0132	10.3	60.8
A0A0H3BWJ2	V-type sodium ATP synthase subunit C	Spy49_0133	14.2	105.7
A0A0H3BX63	V-type sodium ATP synthase subunit G	Spy49_0134	52.9[2]	n.d.[3]
B5XJH3	V-type ATP synthase alpha chain	Spy49_0135	15.3	10.5
B5XJH4	V-type ATP synthase beta chain	Spy49_0136	12.7	14.5
B5XJH5	V-type ATP synthase subunit D	Spy49_0137	64.4	563.9[2]

Figure S4. Expression profiles of the coordinately regulated V-type ATP synthase subunits during cultivation in either THY medium (THY), CDM with fructose (CDMF) or CDM without a carbon source (CDM-). The proteins were significantly expressed only during stationary phase in THY. Results from experiment 1 **(A)** and experiment 2 **(B)** are shown. Protein identities and the maximal fold change are indicated in the table **(C)**.

1) maximal fold change is the highest abundance difference between two growth conditions of the experiment;
2) protein was identified by a single unique peptide;
3) protein was not identified in this experiment.

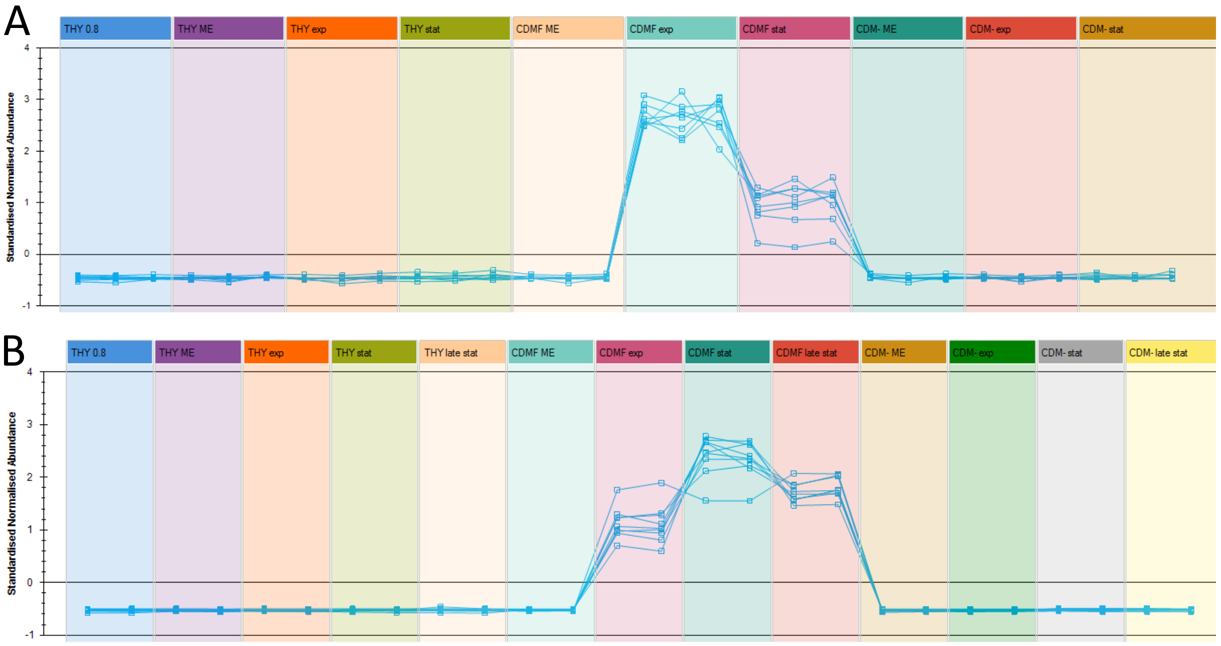

C

UniProt Acc.	Protein name	Gene	Max. FC[1]	
			Exp. 1	Exp. 2
A0A0H3BX93	Shikimate 5-dehydrogenase	Spy49_0450	16.4	182.5
A0A0H3BXU8	AP_endonuc_2 domain-containing protein	Spy49_0451	17.0	33.3
A0A0H3BZ61	F420_ligase domain-containing protein	Spy49_0453	79.0	388.7
A0A0H3BZI2	Uncharacterized protein	Spy49_0454	66.5	37.9
A0A0H3BW88	Archaeal S-adenosylmethionine synthetase	Spy49_0455	89.4	169.7
A0A0H3BX98	Uncharacterized protein	Spy49_0456	74.3	55.1
A0A0H3BXV2	Glyco_trans_2-like domain-containing protein	Spy49_0457	42.1	344.2
A0A0H3BZI9	UDP-glucose 6-dehydrogenase	Spy49_0459	17.5	21.4
A0A0H3BW93	Putative efflux protein	Spy49_0460	1994.7[2]	64.4

Figure S5. Expression profiles of a group of fructose-induced proteins during cultivation in either THY medium (THY), CDM with fructose (CDMF) or CDM without a carbon source (CDM-). The proteins were significantly expressed only in CDMF. The results from experiment 1 **(A)** and experiment 2 **(B)** indicate faster adaption to fructose utilization in experiment 1. Protein identities and the maximal fold change are indicated in the table **(C)**.

[1)] maximal fold change is the highest abundance difference between two growth conditions of the experiment; [2)] protein was identified by a single unique peptide.

A

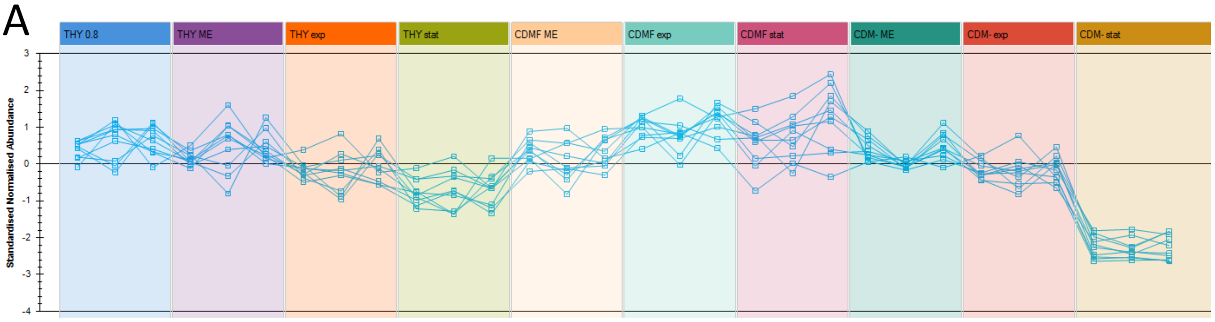

B

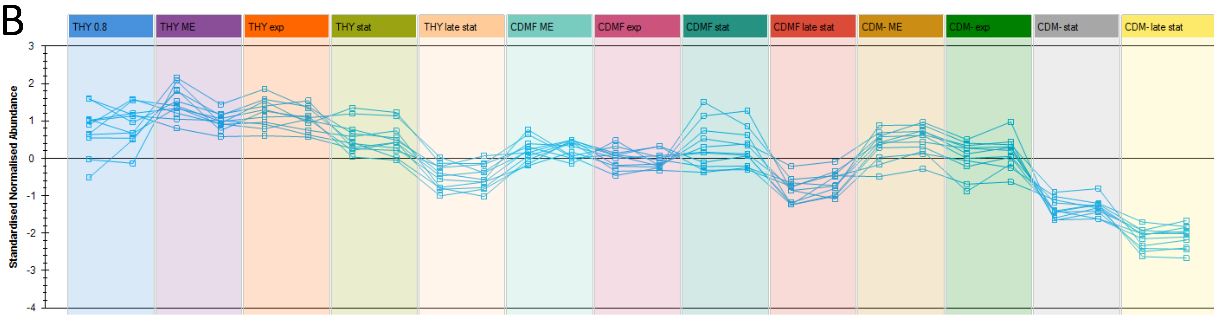

C

UniProt Acc.	Protein name	Gene	Max. FC[1]	
			Exp. 1	Exp. 2
B5XIJ1	30S ribosomal protein S9	Spy49_1595c	<2.0[2]	2.2
A0A0H3BXD5	30S ribosomal protein S12	Spy49_0230	<2.0[2]	2.0
B5XKW7	30S ribosomal protein S16	Spy49_0659	<2.0[2]	2.2
B5XK61	50S ribosomal protein L11	Spy49_0387	<2.0[2]	2.1
B5XJ55	50S ribosomal protein L15	Spy49_0067	2.2	2.3
B5XJ47	50S ribosomal protein L24	Spy49_0058	2.2	5.3
A0A0H3BZK2	50S ribosomal protein L27	Spy49_0645	2.9	3.1
B5XIF6	50S ribosomal protein L28	Spy49_1558c	**5.2**	**7.9**
B5XJ44	50S ribosomal protein L29	Spy49_0055	2.0	3.8
B5XKL7	50S ribosomal protein L31 type B	Spy49_0552c	2.4	3.2
B5XJ84	50S ribosomal protein L32	Spy49_1770	4.5[3]	n.d.[4]
A0A0H3C214	50S ribosomal protein L33	Spy49_1771	2.9	n.d.[4]
B5XKT9	50S ribosomal protein L35	Spy49_0630	**8.8**	**9.2**
B5XJ59	50S ribosomal protein L36	Spy49_0071	5.0[3]	n.d.[4]

Figure S6. Expression profiles of selected ribosomal proteins during cultivation in either THY medium (THY), CDM with fructose (CDMF) or CDM without a carbon source (CDM-). Results from experiment 1 **(A)** and experiment 2 **(B)** are shown. Protein identities and the maximal fold change are indicated in the table **(C)**.

1) maximal fold change is the highest abundance difference between two growth conditions of the experiment;
2) FC was below 2.0 in experiment 1, the corresponding expression profiles are not shown;
3) protein was identified by a single unique peptide;
4) protein was not identified in this experiment.

A

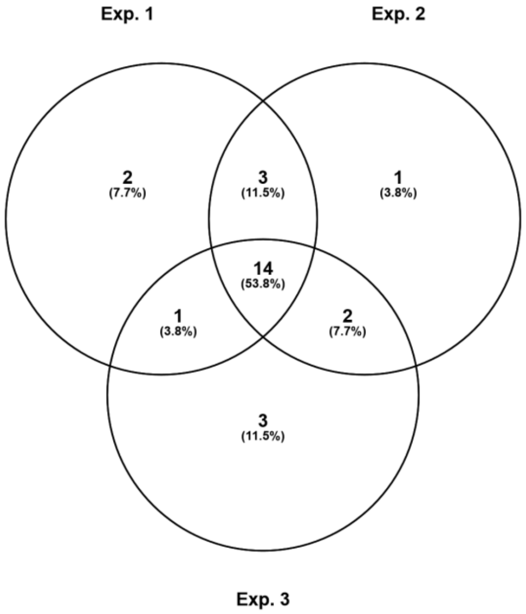

B

UniProt Acc.	Protein name	Gene	Mean abundance[1]	Rank[2] Exp. 1	Rank Exp. 2	Rank Exp. 3
A0A0H3BY55	PASTA kinase SP-STK	Spy49_1257c	3,098,001	1	1	1
A0A0H3BZ18	Cell-division initiation protein	*divIVA*	814,752	2	3	2
A0A0H3BYP4	Phosphoglucosamine mutase	*glmM*	779,102	3	2	5
A0A0H3BZL9	PTS system, fructose-specific IIABC component	*fruA*	179,970	6	4	3
A0A0H3BZC1	Uncharacterized protein	Spy49_0377	177,597	4	5	13
A0A0H3BZD6	Putative PTS system enzyme II	Spy49_1737c	120,096	5	10	4
B5XMJ7	Cell cycle protein GpsB	*gpsB*	102,556	7	7	10
B5XI23	Protein translocase subunit SecA	*secA*	92,742	12	11	6
B5XKI1	Elongation factor Tu	*tuf*	90,657	8	6	16
A0A0H3BYM9	Glyceraldehyde-3-phosphate dehydrogenase	*plr*	87,956	9	9	11
A0A0H3BZR7	Mid-cell-anchored protein Z	*mapZ*	74,843	13	15	9
B5XJ02	UPF0297 protein	Spy49_1751c	70,210	19	14	8
A0A0H3BZ23	Cell division protein FtsZ	*ftsZ*	56,870	14	17	18
A0A0H3C2P8	Uncharacterized protein	Spy49_1748c	55,877	16	20	15

[1] The mean abundance was calculated from the abundances of the phosphoproteins in the three experiments

[2] Rank indicates the order within the 20 most abundant phosphoproteins in each experiment

Figure S7. Quantitatively predominant phosphoproteins in the exponential growth phase (OD_{600} = 0.8) in THY in experiments 1-3. The amounts of all phosphopeptides belonging to a protein were summed up without considering the phosphorylation site, i.e. phosphopeptides with ambiguous phosphosite localization were also taken into account. Phosphopeptide abundances were not normalized to protein levels. From each of the three experiments, the 20 phosphoproteins with the highest abundances were compared **(A)**. The 14 overlapping phosphoproteins are listed in the Table **(B)**.

A

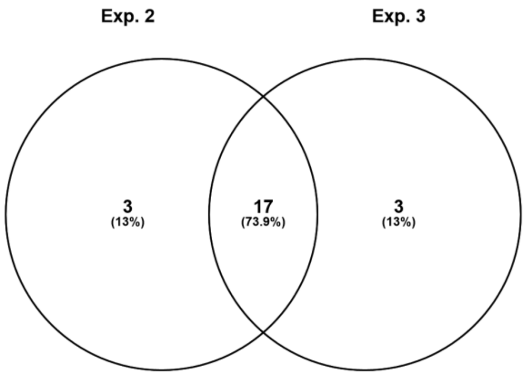

B

UniProt Acc.	Protein name	Gene	Mean abundance[1]	Rank[2] Exp. 2	Rank Exp. 3
A0A0H3BYP4	Phosphoglucosamine mutase	*glmM*	1,074,445	2	3
A0A0H3BYW2	EIICB-Lac lacE	*lacE*	1,071,128	3	2
A0A0H3BZD6	Putative PTS system enzyme II	Spy49_1737c	1,055,155	5	1
B5XKI1	Elongation factor Tu	*tuf*	1,040,779	1	4
B5XIW7	Chaperonin GroEL	*groEL*	819,809	6	5
A0A0H3BY55	PASTA kinase SP-STK	Spy49_1257c	798,559	4	7
A0A0H3BZL9	PTS system, fructose-specific IIABC component	*fruA*	712,445	9	6
A0A0H3BZJ0	Formate acetyltransferase	*pfl*	512,288	8	11
B5XJR1	Elongation factor G	*fusA*	458,399	11	9
A0A0H3C124	Putative mannose-specific phosphotransferase system component IID	*manN*	458,143	7	15
A0A0H3BXI0	Uncharacterized protein	Spy49_1001c	393,400	20	10
B5XHZ0	Chaperone protein DnaK	*dnaK*	386,629	15	12
A0A0H3BY05	Carbamate kinase	*arcC*	357,525	18	13
A0A0H3BYM9	Glyceraldehyde-3-phosphate dehydrogenase	*plr*	331,151	14	14
A0A0H3BZX9	PTS system, mannose-specific IIC component	*manM*	307,747	12	18
A0A0H3BZ18	Cell-division initiation protein	*divIVA*	272,167	16	20
A0A0H3BWF1	Protein translocase subunit SecY	*secY*	270,617	19	19

[1] The mean abundance was calculated from the abundances of the phosphoproteins in both experiments

[2] Rank indicates the order within the 20 most abundant phosphoproteins in each experiment

Figure S8. Quantitatively predominant phosphoproteins in the late stationary growth phase in THY in experiments 2 and 3. The amounts of all phosphopeptides belonging to a protein were summed up without considering the phosphorylation site, i.e. phosphopeptides with ambiguous phosphosite localization were also taken into account. Phosphopeptide abundances were not normalized to protein levels. The 20 most abundant phosphoproteins in both experiments were compared **(A)**. The 17 overlapping phosphoproteins are listed in the Table **(B)**.

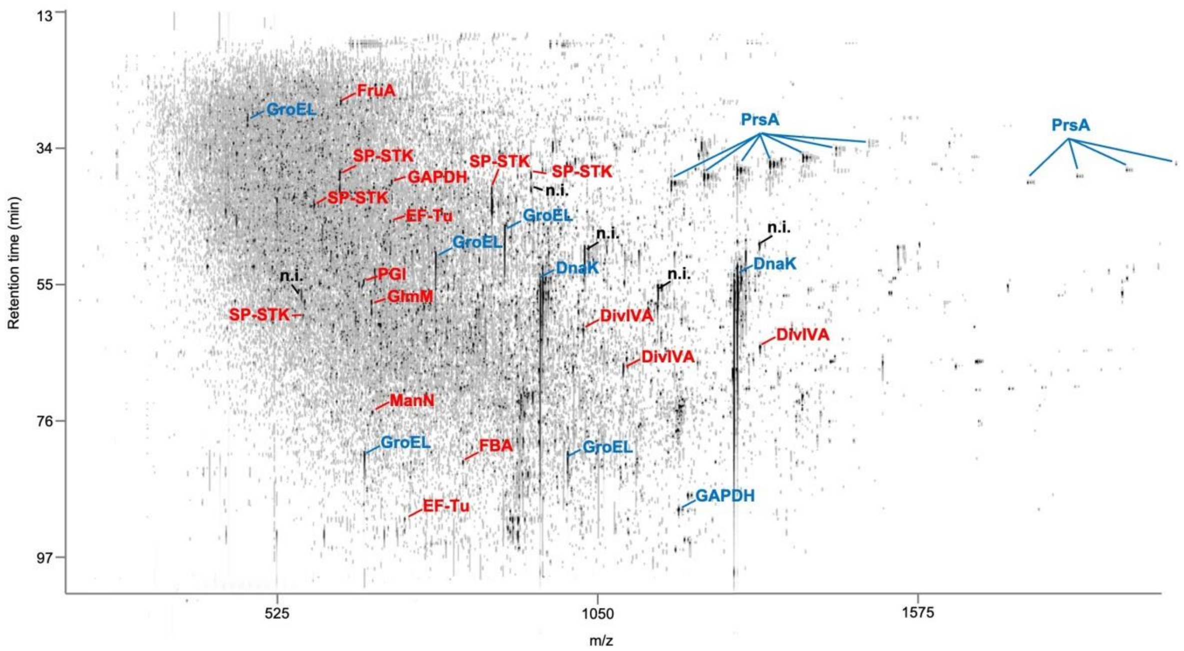

Figure S9. Evaluation of the phosphopeptide enrichment method. A plot of m/z values versus retention time combines all features from the HDMS^{E} measurements of experiment 2. Selected nonphosphorylated peptide ions (labeled blue) and phosphopeptide ions (labeled red) are highlighted. DnaK - chaperone protein DnaK; GroEL – chaperonin GroEL; GAPDH - glyceraldehyde-3-phosphate dehydrogenase; PrsA – foldase protein PrsA; SP-STK - non-specific serine/threonine protein kinase; DivIVA - cell-division initiation protein; EF-Tu - Elongation factor Tu; FruA - PTS system fructose-specific IIABC component; FBA - fructose-bisphosphate aldolase class II; PGI - glucose-6-phosphate isomerase; GlmM - phosphoglucosamine mutase; ManN - putative mannose-specific phosphotransferase system component IID; n.i. - not identified.

To evaluate the specificity of phosphopeptide enrichment and the efficiency of identification, we inspected the plot of m/z versus retention time combining all HDMS^{E} data of the second experiment. The most abundant peptide ions derived from one large nonphosphorylated peptide (MYEQAAAAQQAAQGAEGAQANDSANNDDVVDGEFTEK) of chaperone protein DnaK. Nonphosphorylated peptides of the chaperonin GroEL were also highly abundant. Among phosphopeptides, prominent ion signals derived from the PASTA kinase SP-STK and cell-division initiation protein (DivIVA). However, many abundant signals were not identified including a striking pattern of ions with decreasing retention time at increasing m/z. It comprises a series of seven triply charged peptides, each differing by a mass difference of 162 Da, all of which could be assigned to the C-terminal peptide of the foldase protein PrsA (PrtM1) by manual analyses of fragment spectra (Figure S10).

Moreover, an error-tolerant Mascot search revealed frequently occurring lysine phosphoglycerylation (PGK) [42,43] among the enriched phosphopeptides. The specificity of the phosphopeptide enrichment was evaluated for the second experiment based on both the number of unique peptides and their quantitative ratios. Of the total of 3581 peptides identified, the proportion of S/T/Y-phosphorylated peptides was 26%, while 63% were not phosphopeptides and 11% of peptides were modified by lysine phosphoglycerylation. Label-free quantification revealed percentages of S/T/Y-phosphorylated peptides, nonphosphorylated peptides, and PGK-modified peptides of 24%, 73%, and 3%, respectively.

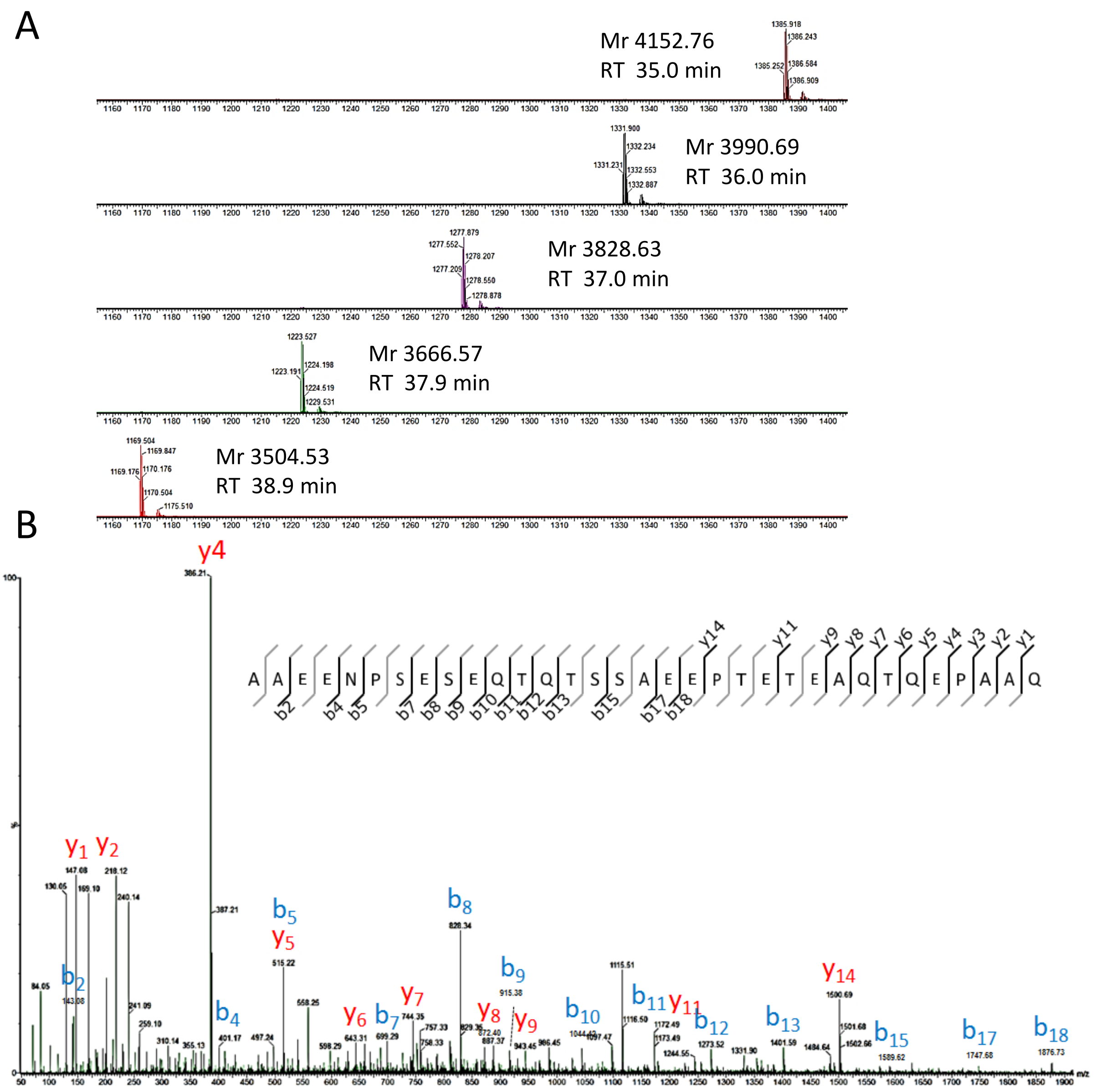

Figure S10. Evidence that foldase protein PrsA (PrtM1) is a glycoprotein. A series of triply charged C-terminal peptide ions, each differing by a mass of 162 Da, indicate glycosylation **(A)**. Representative fragment spectrum of the C-terminal peptide ion of foldase protein PrsA. The site of glycan modification could not be identified, probably due to the lability of the glycosidic bond **(B)**.

A mass increase of 162 Da indicates a sugar moiety. However, the mass spectra did not provide further information about the exact location and nature of the modification. Foldase PrsA is a membrane-bound lipoprotein with peptidyl-prolyl cis-trans isomerase activity that supports folding of exported proteins and contributes to bacterial virulence. Its identification as a putative glycoprotein may prompt further studies that could provide new insights into the virulence mechanisms of *S. pyogenes*.

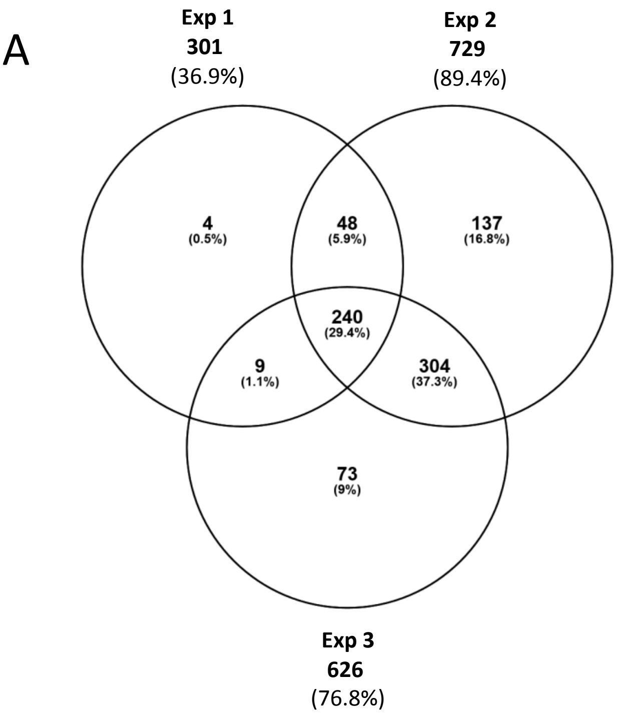

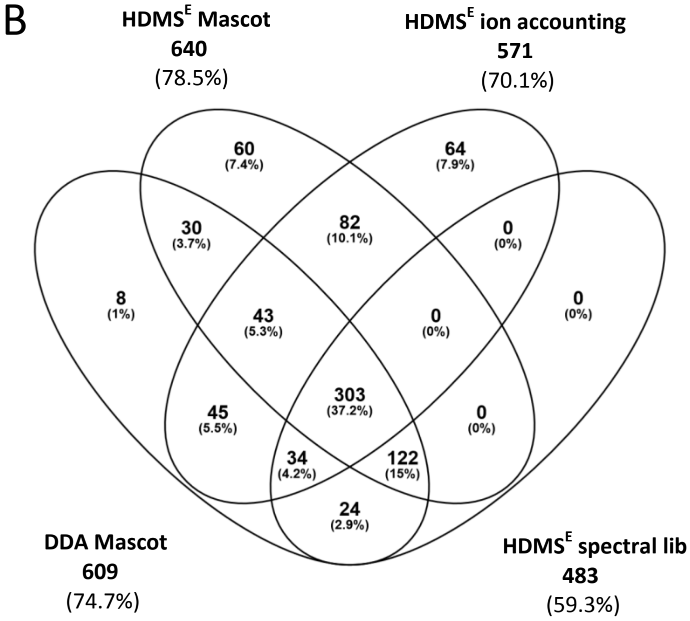

Figure S11. Venn diagrams showing numbers and percentages of high-confidence phosphorylation sites identified in each of the three experiments **(A)** and the contribution of different data acquisition and search strategies to the identification of 815 high-confidence phosphorylation sites **(B)**.

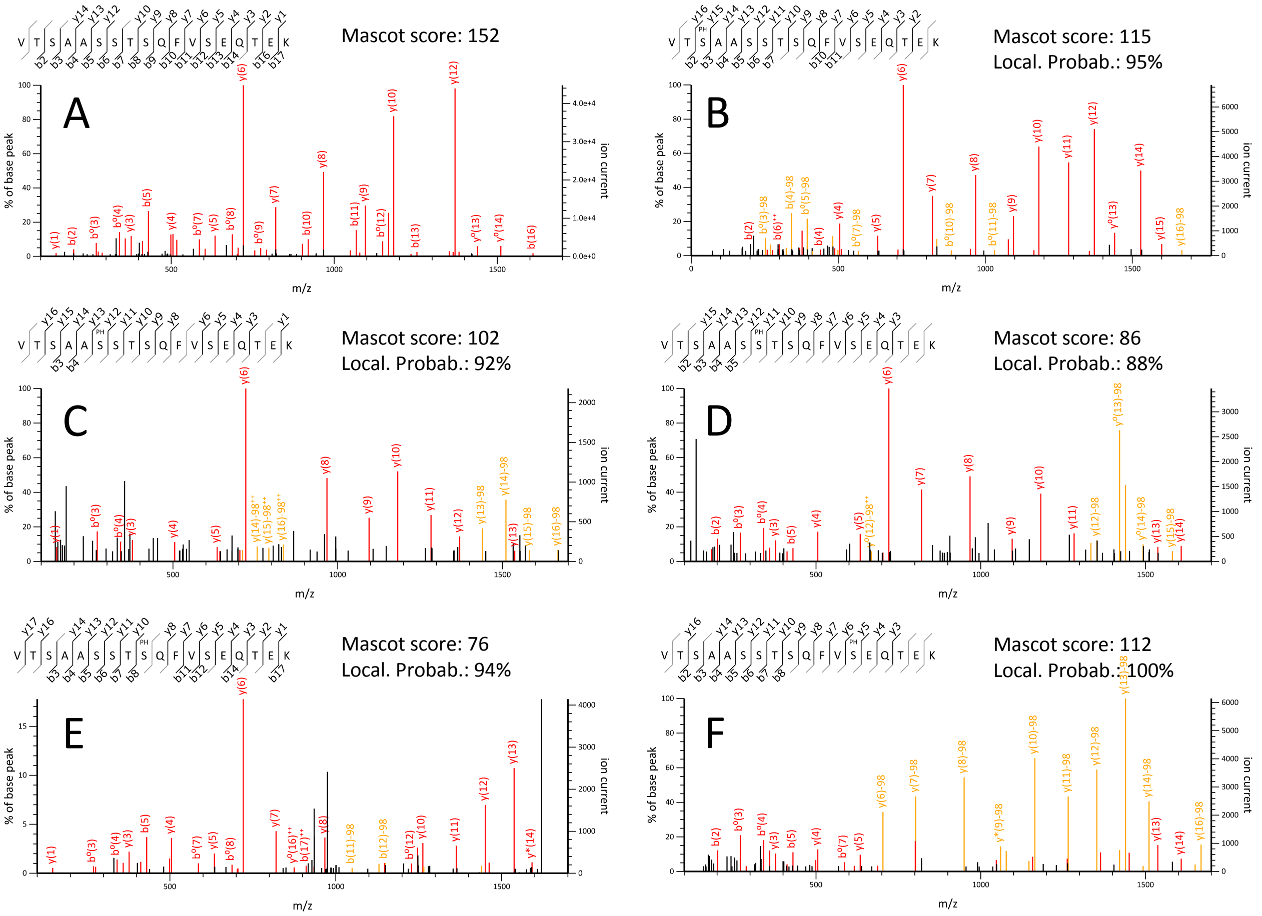

G

MEKTQIKSTLTYDDKVIEKIVGHALENVGGLLAVTGGFFSNIKNNLVNSESVTDGVSVEV
GSKEVAVDLAIIVEYGKDIPAIAE**S**IKAIVSQNVD**S**MTHLKVVEVNVNVVDIRTKEEHEA
A**S**V**T**VQDRVT**S**AA**SS**T**S**QFV**S**EQTEKLKDTI**S**DTVN**S**DEAAK

129 146

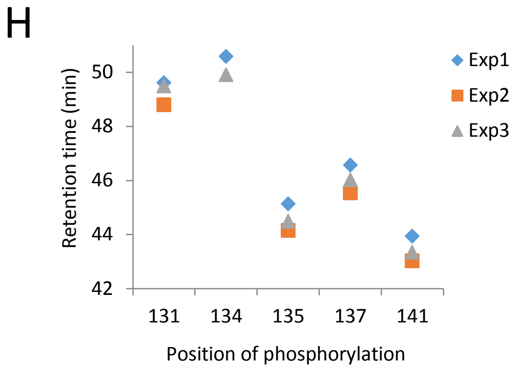

Figure S12. Detection of phosphopeptide positional isomers of the peptide VTSAASSTSQFVSEQTEK of the general stress protein, Gls24 family (Spy49_1001c). MS/MS spectra of doubly protonated peptides were acquired by HDMS^E and identified using Mascot. The peptide was found nonphosphorylated **(A)**, and phosphorylated at S131 **(B)**, S134 **(C)**, S135 **(D)**, S137 **(E)**, and S141 **(F)**. All identified phosphosites of the protein are indicated in bold red in the amino acid sequence of the Spy49_1001c protein. The multiply phosphorylated peptide 129-146 is highlighted in grey **(G)**. Separation of the phosphopeptide isomers by their retention time is shown in **(H)**.

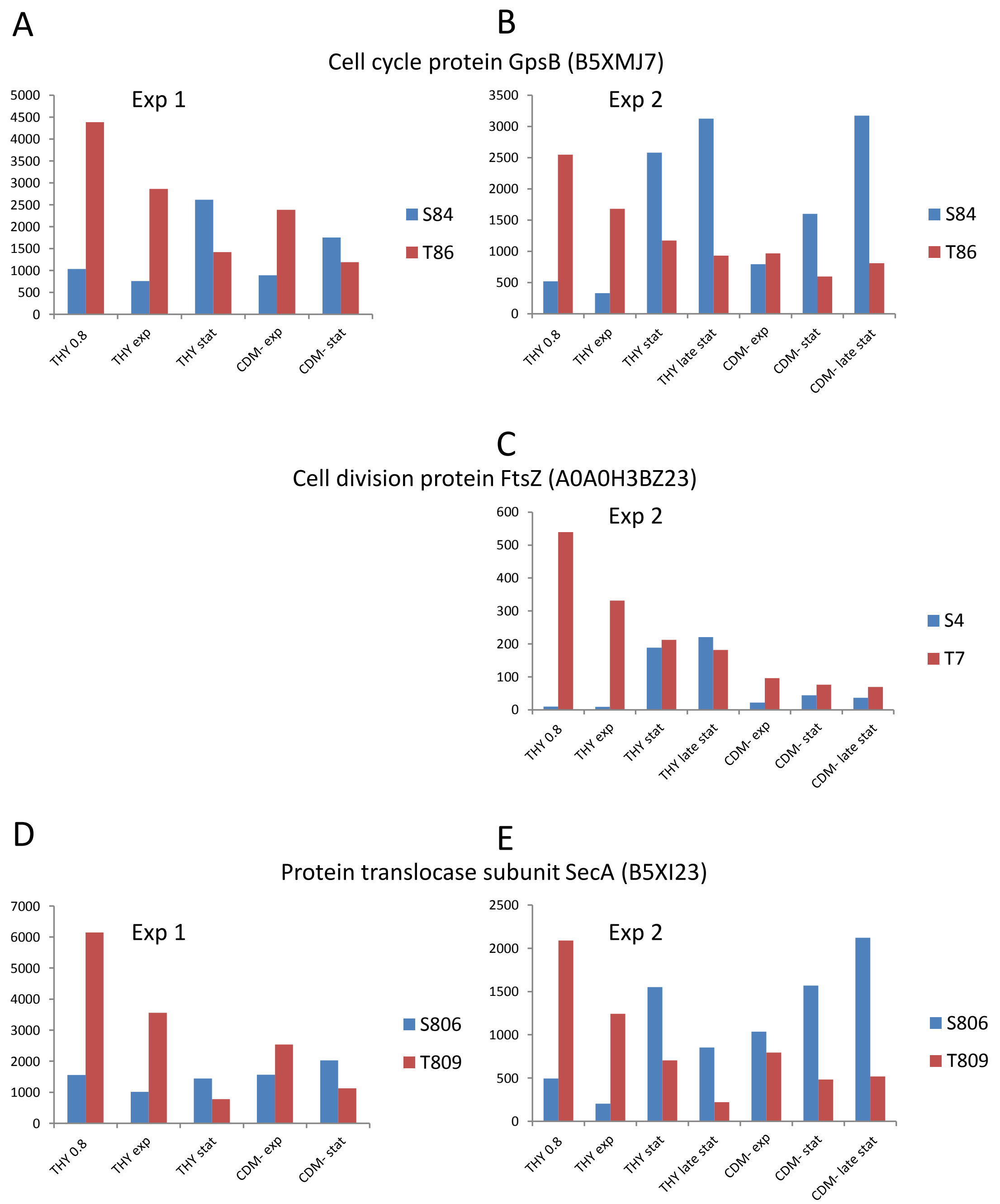

Figure S13. Growth phase-dependent phosphorylation of nearby threonine and serine residues located on the same tryptic peptide. **(A,B)** Phosphorylation dynamics of cell cycle protein GpsB in the first **(A)** and second **(B)** experiment. **(C)** Phosphorylation dynamics of cell division protein FtsZ in the second experiment. The phosphosites were not quantified in the first experiment. **(D,E)** Phosphorylation dynamics of protein translocase subunit SecA in the first **(D)** and second **(E)** experiment.

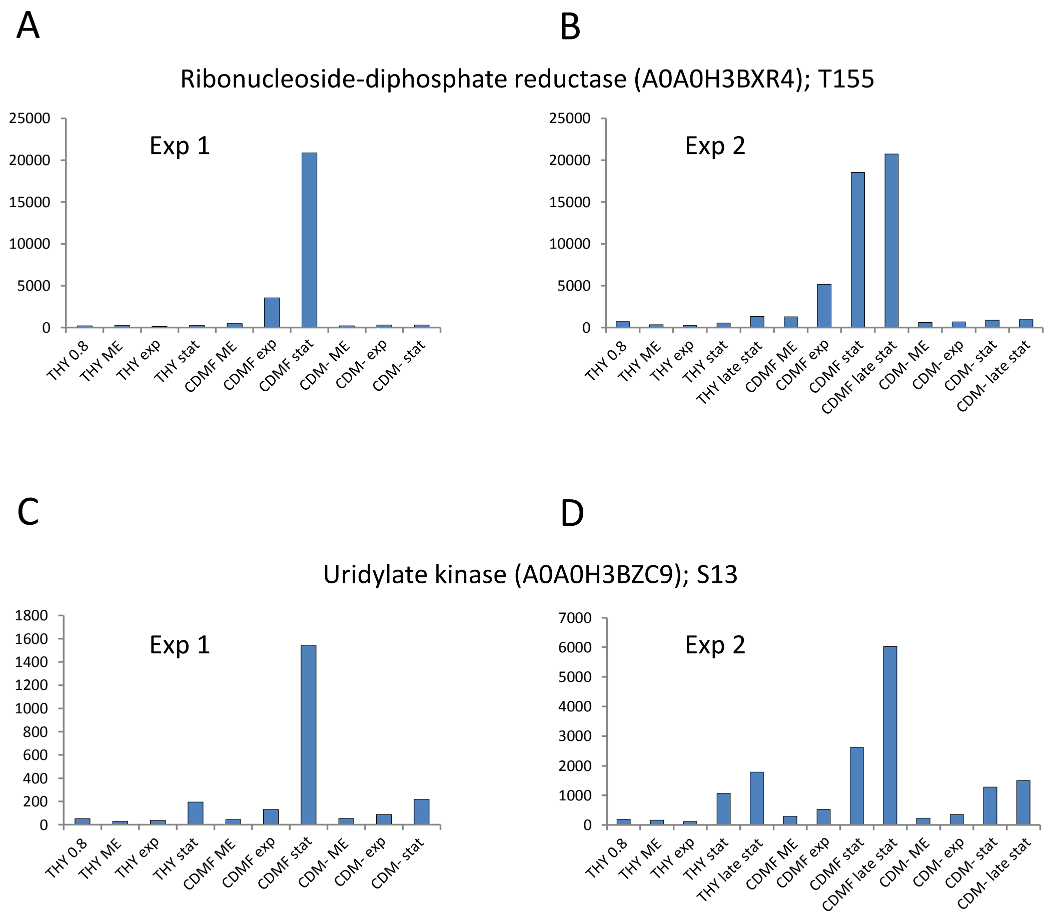

Figure S14. Increased phosphorylation events during cultivation in CDMF. **(A,B)** Phosphorylation dynamics of T155 of ribonucleoside-diphosphate reductase in the first **(A)** and second **(B)** experiment. **(C,D)** Phosphorylation dynamics of S13 of uridylate kinase in the first **(C)** and second **(D)** experiment.

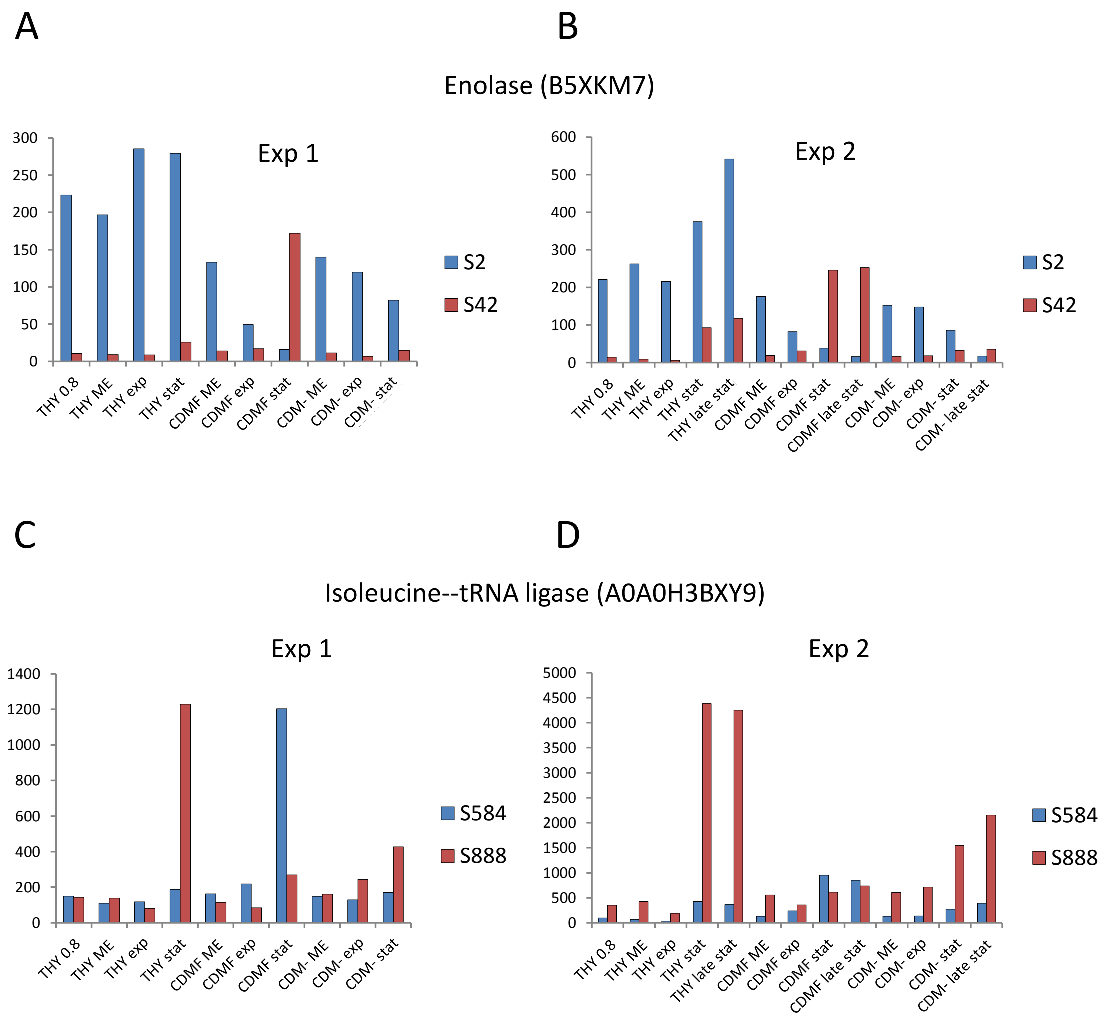

Figure S15. Phosphorylation dynamics of enolase and isoleucine-tRNA ligase at different culture conditions. **(A,B)** Phosphorylation dynamics of enolase in the first **(A)** and second **(B)** experiment. **(C,D)** Phosphorylation dynamics of isoleucine-tRNA ligase in the first **(C)** and second **(D)** experiment.

```
CLUSTAL O(1.2.4) multiple sequence alignment


tr|Q836G2|Q836G2_ENTFA              --MANLVYSPKDILQKEFKTKMMNGYDPIEVDEFLDNVIKDYEAYNKELLSLQEENSRLM  58
sp|P0CI74|GPSB_BACSU                MLADKVKLSAKEILEKEFKTG-VRGYKQEDVDKFLDMIIKDYETFHQEIEELQQENLQLK  59
sp|B5XMJ7|GPSB_STRPZ                --MTSIIYSPKDIFEQEFKTS-MRGFDKKEVDEFLDNVIKDYENFNAQIEALKAENEALK  57
tr|A0A0H3MTG8|A0A0H3MTG8_STRS4      --MASIKFTTKDIFEQDFKIG-FRGYDQDEVNDFLDDIMKDYDAYEAIIKELKGEIARLK  57
sp|Q04M95|GPSB_STRP2                --MASIIFSAKDIFEQEFGRE-VRGYNKVEVDEFLDDVIKDYETYAALVKSLRQEIADLK  57
                                        .:  : *:*::::*    ..*:.  :*:.*** ::***: :   :  *: *   *

tr|Q836G2|Q836G2_ENTFA              AKLDQLSKAQPTPRV---AQEVPKSAAVTNFDILKRLSNLEREVFGKKLDETPSTPVTPS  115
sp|P0CI74|GPSB_BACSU                KQLEEASKKQP------------VQSNTTNFDILKRLSNLEKHVFGSKLYD---------  98
sp|B5XMJ7|GPSB_STRPZ                KAKYQARNTVSATVQQPVPQPTRVAQSATNFDILKRISKLEKEVFGKQIIE---------  108
tr|A0A0H3MTG8|A0A0H3MTG8_STRS4      AQAANSPKTTLPTEESNDVLRTERPSSATNFDILRRLNRLEKEVFGKQIVQDQE------  111
sp|Q04M95|GPSB_STRP2                EELTRKPKPSP---VQAEPLEAAITSSMTNFDILKRLNRLEKEVFGKQILDNSDF-----  109
                                        .  :                    ******:*:..**:.***.:: :

tr|Q836G2|Q836G2_ENTFA              APSMPAEPANHDVDNAQTRQF        136
sp|P0CI74|GPSB_BACSU                ---------------------        98
sp|B5XMJ7|GPSB_STRPZ                ---------------------        108
tr|A0A0H3MTG8|A0A0H3MTG8_STRS4      ---------------------        111
sp|Q04M95|GPSB_STRP2                ---------------------        109
```

Figure S16. Protein sequence alignment of GpsB indicates conserved phosphorylation sites. Phosphosites that have been identified as targets of the respective PASTA kinases in *E. faecalis* (ENTFA) [50], *B. subtilis* (BACSU) [65], *S. suis* (STRS4) [46], and *S. pneumoniae* (STRP2) [44] are highlighted in green. T66 and T86 of *S. pyogenes* (STRPZ), which were more strongly phosphorylated during growth than in the stationary phase, are highlighted in red. Other phosphorylation sites of the GpsB protein of *S. pyogenes* found in our analysis are highlighted in turquoise.
