# Supplementary figures and images for: Dynamic Protein Phosphorylation in Streptococcus pyogenes during Growth, Stationary Phase, and Starvation
Source: Microorganisms. 2024 Mar 20;12(3):621. doi: 10.3390/microorganisms12030621 (PMC10975399; doi:10.3390/microorganisms12030621)

Experiment 1

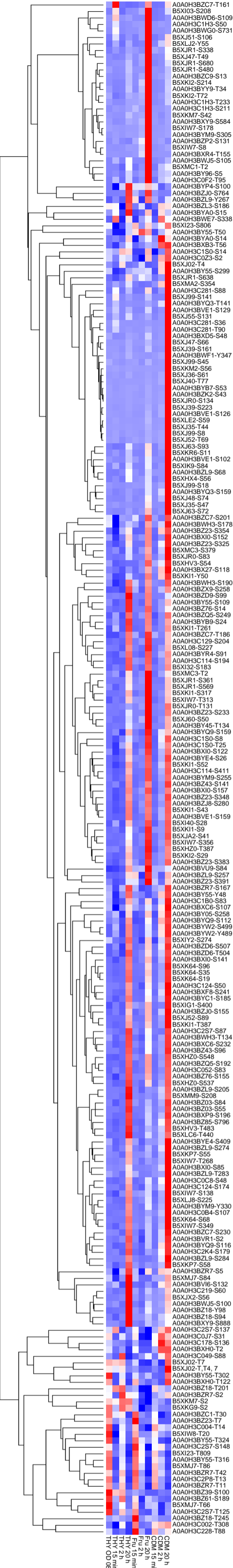

Data Distribution

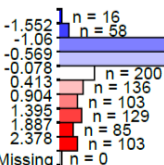

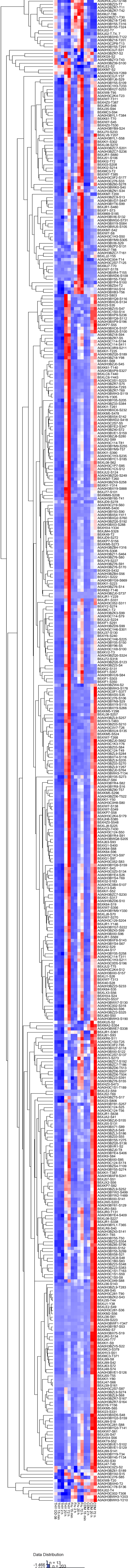

Data Distribution

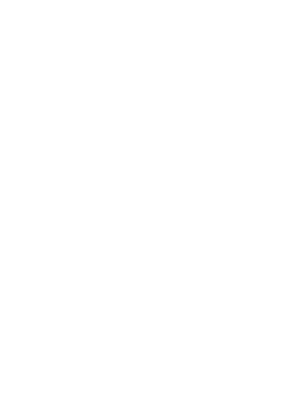

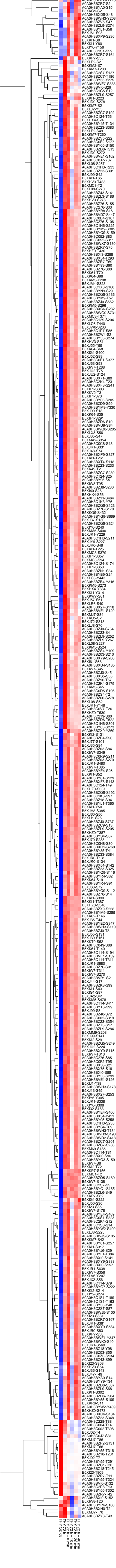

Data Distribution

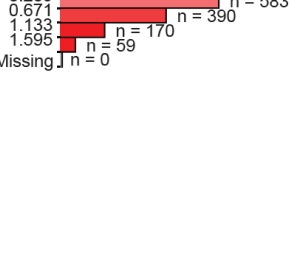

Supplement: Supplementary file 1 [file microorganisms-12-00621-s001.zip › Supplementary Data Sheet S1.pdf]
